# WSSV exploits AMPK to activate mTORC2 signaling for proliferation by enhancing aerobic glycolysis

**DOI:** 10.1038/s42003-023-04735-z

**Published:** 2023-04-03

**Authors:** Peng Zhang, Hai-Jing Fu, Li-Xia Lv, Chen-Fei Liu, Chang Han, Xiao-Fan Zhao, Jin-Xing Wang

**Affiliations:** 1 Shandong Provincial Key Laboratory of Animal Cells and Developmental Biology, School of Life Sciences, Shandong University, 266237 Qingdao, Shandong China; 2 State Key Laboratory of Microbial Technology, Shandong University, 266237 Qingdao, Shandong China

**Keywords:** Immune evasion, Infection, Pathogens, Innate immunity

## Abstract

AMPK plays significant roles in the modulation of metabolic reprogramming and viral infection. However, the detailed mechanism by which AMPK affects viral infection is unclear. The present study aims to determine how AMPK influences white spot syndrome virus (WSSV) infection in shrimp (*Marsupenaeus japonicus*). Here, we find that AMPK expression and phosphorylation are significantly upregulated in WSSV-infected shrimp. WSSV replication decreases remarkably after knockdown of *Ampkα* and the shrimp survival rate of AMPK-inhibitor injection shrimp increases significantly, suggesting that AMPK is beneficial for WSSV proliferation. Mechanistically, WSSV infection increases intracellular Ca^2+^ level, and activates CaMKK, which result in AMPK phosphorylation and partial nuclear translocation. AMPK directly activates mTORC2-AKT signaling pathway to phosphorylate key enzymes of glycolysis in the cytosol and promotes expression of Hif1α to mediate transcription of key glycolytic enzyme genes, both of which lead to increased glycolysis to provide energy for WSSV proliferation. Our findings reveal a novel mechanism by which WSSV exploits the host CaMKK-AMPK-mTORC2 pathway for its proliferation, and suggest that AMPK might be a target for WSSV control in shrimp aquaculture.

## Abstract (web summary)

AMPK and its downstream signaling pathways are implicated in the white spot syndrome virus (WSSV) response of the shrimp Marsupenaeus japonicus, with WSSV using this pathway to promote its proliferation.

## Introduction

Adenosine 5′-monophosphate (AMP)-activated protein kinase (AMPK) is an intracellular energy state sensor and a glucose sensor in eukaryotic cells^1^. AMPK forms a heterotrimer complex consisting of three subunits, the α catalytic and β and γ regulatory subunits^2,3^. Phosphorylation of AMPKα at Thr^172^ within the activation loop of the kinase domain is the primary marker and necessary condition for AMPK activation^4,5^. The carbohydrate-binding module (CBM) domain of the β subunit enables AMPK to bind to glycogen surface particles^2^. The γ subunit contains four tandem cystathionine beta-synthase (CBS) repeat domains that contain regulatory nucleotide AMP, ADP, and ATP binding sites^6,7^. AMPK activation reprograms the metabolic process by reducing anabolism and increasing catabolism to restore energy balance in animals^8,9^.

AMPK is a master regulator of cell survival, autophagy, stress responses, metabolic reprogramming, and mitochondrial homeostasis^10^. AMPK also play double-edged roles (beneficial or detrimental for pathogens) in the regulation of host antimicrobial defenses during viral infection^11,12^. For example, AMPK was activated by Hepatitis B virus in HepG2.2.15 cells and decreased HBV replication through the promotion of autophagic degradation^13^. Rift Valley fever virus (RVFV) infection activates AMPK, leading to the phosphorylation and inhibition of acetyl-CoA carboxylase to decrease fatty acid synthesis, which restricts RVFV infection^14^. Activation of AMPK restricts Coxsackie virus B3 (CVB3) replication by inhibiting lipid accumulation^15^. By contrast, in some cases, virus-induced AMPK activation benefits viral replication. Respiratory syncytial virus (RSV) infection induces autophagy through reactive oxygen species (ROS) generation and activation of the AMPK-mechanistic target of rapamycin (mTOR) signaling pathway to promote viral replication^16^. Avian reovirus (ARV) infection increases the phosphorylation of AMPK, and facilitates MAP kinase kinase (MKK) 3/6, mitogen associated protein kinase (MAPK), and p38 signaling, which are required for virus replication^17^. Infection of porcine reproductive and respiratory syndrome virus (PRRSV) induces the activation of the AMPK-acetyl-CoA carboxylase 1 (ACC1) pathway and fatty acid synthesis, both of which are essential for PRRSV replication^18^.

AMPK and mTOR pathways are interrelated in the cell regulation of anabolism and catabolism. mTOR forms two structurally and functionally distinct complexes, mTOR complex 1 (mTORC1) and mTORC2. AMPK activation is known to suppress mTORC1 signaling and inhibits anabolic processes, while promoting catabolic processes, such as autophagy, to restore energy homeostasis^19,20^. In addition, AMPK directly activates mTORC2 in energy stress, by an as yet unknown the mechanism^21,22^. A recent study found that AMPK phosphorylates mTOR at Ser^1261^ and possibly other sites in both mTOR and rapamycin-insensitive companion of mTOR (Rictor), which are the core subunits of mTORC2, leading to mTORC2 activation and increased cell survival in response to energy stress in mammalian cells^23,24^. In shrimp, it is reported that white spot syndrome virus (WSSV) achieves successful replication by altering host metabolic pathways via phosphatidylinositol-4,5-bisphosphate 3-kinase (PI3K)-protein kinase B (AKT)-mTOR signaling, and utilizes the signal pathway-regulated Warburg effect to counter the ROS produced by the host in response to WSSV infection^25,26^. However, it is unclear the exact functions of mTORC1 and mTORC2 in the process.

AMPK can be activated by three upstream kinases: Liver kinase B1 (LKB1), calcium/calmodulin-dependent protein kinase kinase β (CaMKKβ), and transforming growth factor β-activated kinase 1 (TAK1)^27^. The phosphorylation of AMPK by upstream kinases can increase its activity by more than 100 times^28^. Calcium (Ca^2+^) is a ubiquitous intracellular messenger in certain important signal transduction processes, including enzyme activation, cell division and differentiation^29^. Excessive cytoplasmic calcium released from the endoplasmic reticulum activates AMPK via CaMKK^30^. Some viruses can utilize the host Ca^2+^-associated pathway to promote their replication^31^. Previous research has shown that intracellular calcium increases after WSSV infection and CaMKK is activated by the increased cytoplasmic Ca^2+^^32^. It is unknown whether CaMKK activates AMPK signaling in shrimp.

The shrimp culture is an important industry in the coastal areas of Asia and the Americas. However, disease prevention and control remain a problem in the industry^33,34^. WSSV is one of the most lethal viruses to shrimp, causing serious economic losses in the industry^35–37^. A previous study reported that mTORC1 and mTORC2 is activated after WSSV infection via unknown regulators during WSSV genome replication stage^25,38^. In our transcriptome analysis, we found that the *Ampk* was upregulated by WSSV infection in the shrimp *Marsupenaeus japonicus*. To investigate the function of AMPK, we performed RNA interference to knock down the expression of *Ampkα* and *Ampkβ* in shrimp and found that the proliferation of WSSV decreased significantly. An AMPK inhibitor (Dorsomorphin 2HCl) or activator (5-Aminoimidazole-4-carboxamide-1-β-d-ribofuranoside (AICAR)) injection verified these results. This finding was unexpected, because AMPK has traditionally been thought to inhibit mTORC1 signaling, and WSSV utilizes the mTORC1 signaling for its replication in shrimp^25^. To determine how viruses manipulate host immune responses to support their own growth and survival is essential to identify mechanisms of pathogenicity and host adaptation^39^, and this kind of study can provide novel strategies for viral induced disease control. Therefore, in the present study, we aimed to clarify the mechanism by which AMPK benefits viral proliferation. Our data showed that the mechanism comprises AMPK activation of mTORC2 signaling, but not AMPK-mediated suppression of mTORC1 signaling, in response to WSSV infection. We found that WSSV infection induces AMPK phosphorylation via Ca^2+^-CaMKK, and the phosphorylated AMPK activates the mTORC2-AKT axis to promote glycolysis for energy production used in WSSV multiplication in the host cells.

## Results

### The expression and phosphorylation of AMPK are upregulated in shrimp challenged by WSSV

**Fig. 1:**
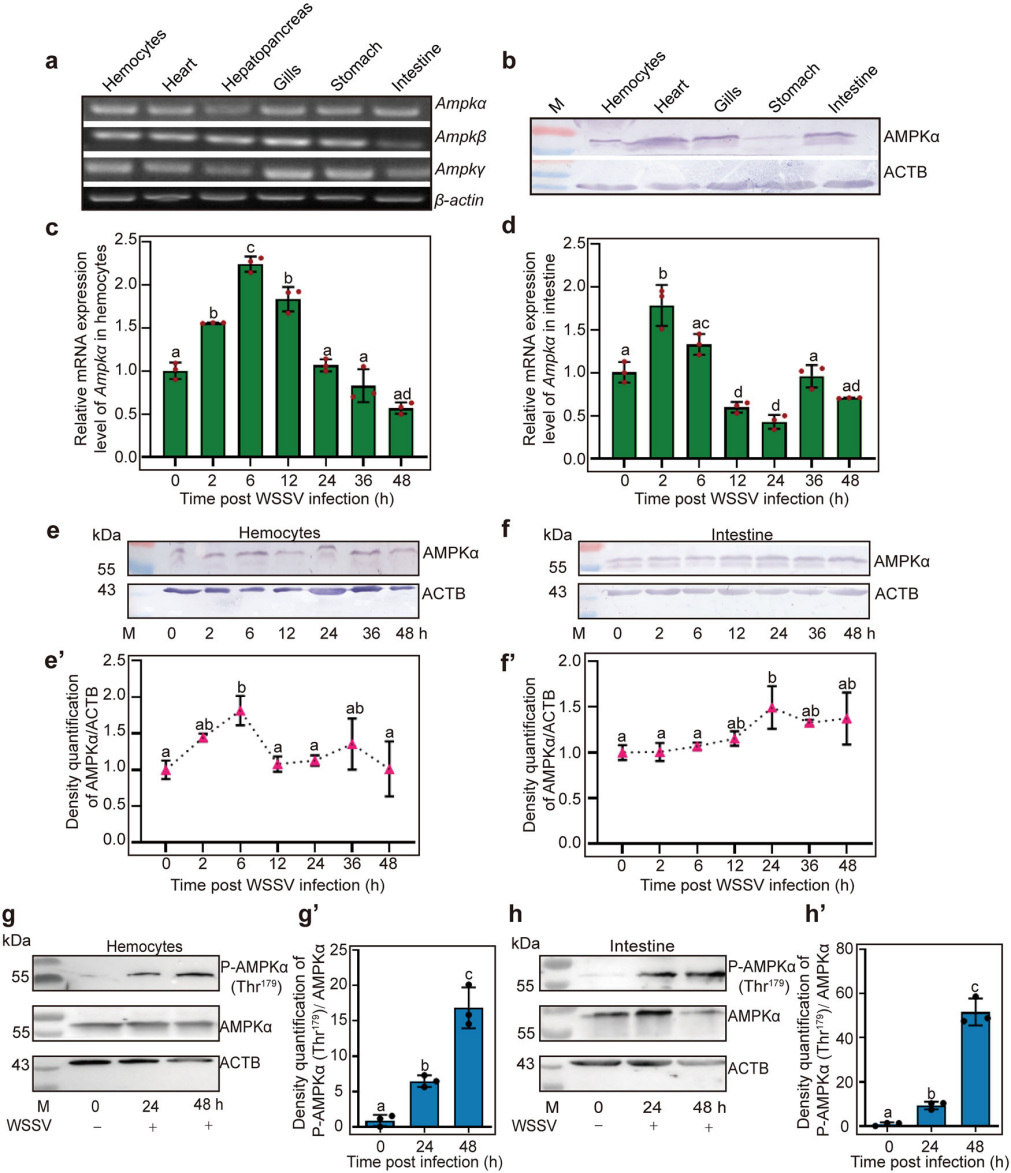
AMPK expression and phosphorylation were upregulated in *M. japonicas* after WSSV challenge. **a** Tissue distribution of *AMPKα/β/γ* in *M. japonicas* at the mRNA level. **b** The distribution of AMPKα proteins in different tissues, detected using western blotting. **c**, **d** The expression patterns of *Ampkα* in hemocytes (**c**) and intestine (**d**) of shrimp challenged by WSSV, analyzed using qPCR. **e**, **f** Expression patterns of AMPKα at the protein level in hemocytes (**e**) and intestine (**f**) of shrimp challenged by WSSV, analyzed via western blotting. **e′**, **f′** is the statistical analysis of **e**, **f** based on three replicates. **g**, **h** AMPK phosphorylation in hemocytes (**g**) and intestine (**h**) were assayed in shrimp at 0, 24, and 48 h post WSSV infection using western blotting with an antibody recognizing phosphorylated AMPK; ACTB (β-actin) was used as loading control. **g′**, **h′** is the statistical analysis of panel **g**, **h** based on three replicates.

The full-length cDNA sequences encoding the AMPK heterotrimeric complex were obtained from hemocyte and intestine transcriptome sequencing of *M. japonicus*, including AMPK catalytic α subunit (AMPKα), and β and γ regulatory subunits (Supplementary Fig. 1a; GenBank accession nos. OL364937, OL364938, and XP042865543, respectively). The AMPKα subunit contains a typical serine-threonine kinase domain at the N-terminus, the β subunit contains the carbohydrate-binding modules (CBMs), and the γ subunits have four tandem repeats of cystathionine beta-synthase domain (CBS) with regulatory adenine nucleotide-binding sites (Supplementary Fig. 1a). AMPKα was expressed in *E. coli* and purified using GST affinity chromatograph (Supplementary Fig. 1b) and anti-AMPKα polyclonal antibodies were prepared (Supplementary Fig. 1c). The amino acid sequences of AMPKα from *M. japonicus* and other species were aligned, which indicated that AMPKα, especially the AMPKα S_TKc domain, is highly conserved (Supplementary Fig. 2), and suggesting that AMPK proteins have been functionally indispensable throughout evolution. The tissue distribution of the three AMPK subunits was analyzed by RT-PCR, which showed that all three subunits were expressed in hemocytes, heart, hepatopancreas, gills, stomach, and intestines (Fig. 1a). The AMPKα protein was also detected in all tested tissues, as revealed by western blotting analysis (Fig. 1b). We then performed a time course expression analysis of AMPKα/β/γ in hemocytes and intestines using qPCR. The results showed that *Ampkα* (Fig. 1c, d), *Ampkβ* (Supplementary Fig. 3a), and *Ampkγ* (Supplementary Fig. 3b) were upregulated in hemocytes and intestines of shrimp after WSSV challenge. Similar to mRNA expression patterns, the AMPKα protein level was also upregulated in shrimp challenged by WSSV (Fig. 1e, f). The level of phosphorylated AMPK in shrimp after WSSV infection was analyzed using a human anti-p-AMPKα1/α2 (Thr^183/172^) antibody (the phosphorylation site of shrimp AMPKα is Thr^179^), and results showed that phosphorylated AMPKα (Thr^179^) levels increased significantly from 24 to 48 h post WSSV infection (Fig. 1g, h). These results suggested that AMPK is involved in WSSV infection and its increased expression and phosphorylation prompted us to explore the functions of AMPK in shrimp immunity.

### WSSV replication is decreased in *Ampk*-knockdown shrimp infected by WSSV

**Fig. 2:**
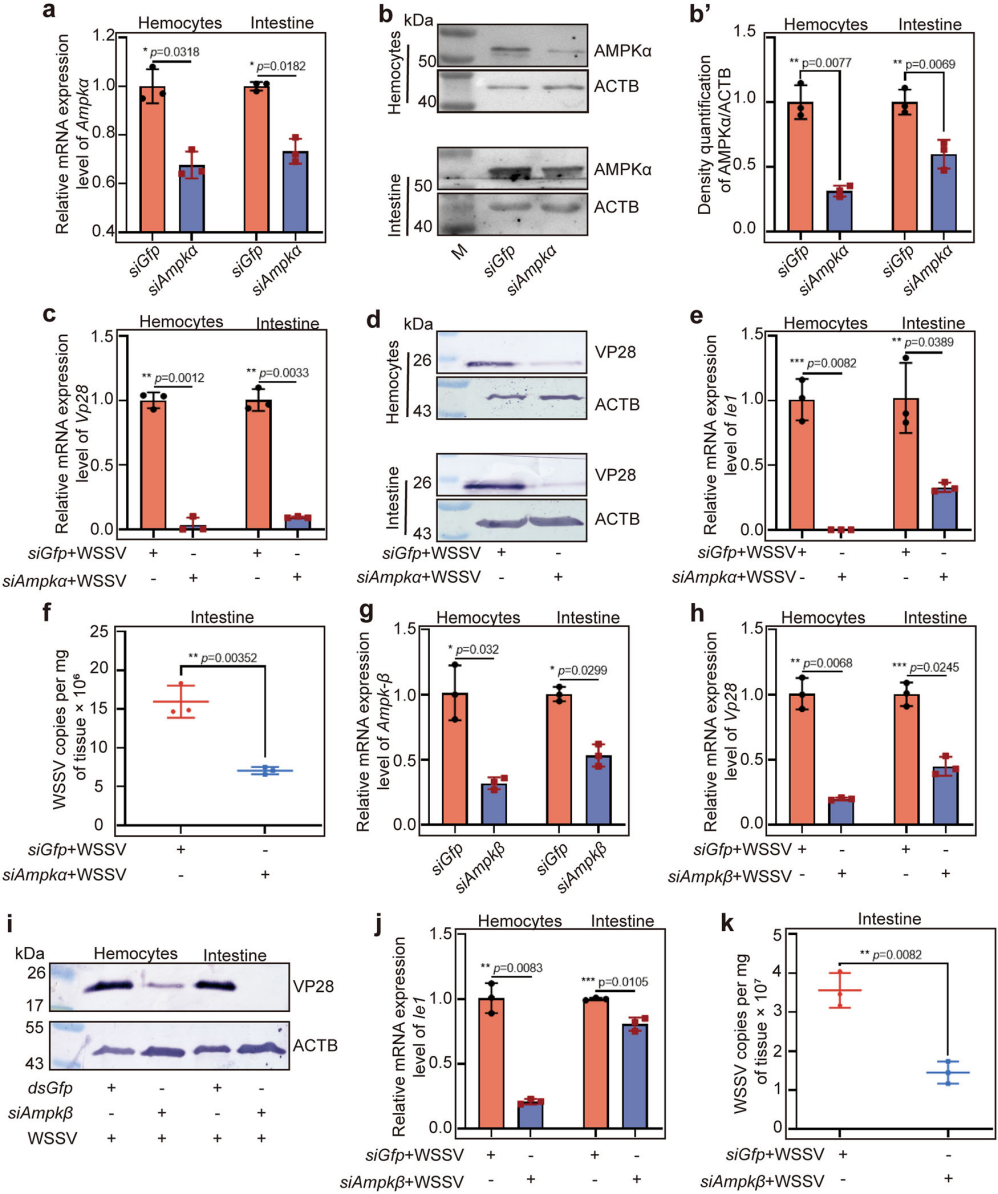
WSSV replication is inhibited in *Ampk*-RNAi shrimp. **a**, **b** Efficiency of *Ampkα* RNAi in hemocytes and intestines of shrimp 36 h post injection of siRNA, analyzed using qPCR (**a**) and western blotting (**b**). **b**′ is the statistical analysis of panel **b** based on three replicates. **c**, **d** Expression levels of *Vp28* mRNA (**c**) and protein (**d**) in hemocytes and intestine of the *siGfp* and *siAmpkα* injection groups challenged by WSSV. **e** Relative expression levels of *Ie1* in hemocytes and intestine of *Ampkα*-RNAi shrimp. **f** copies of WSSV in the intestine of *siGfp* and *siAmpkα* groups after WSSV challenge. **g** RNA interference efficiency of *Ampkβ* in hemocytes and intestine of shrimp 36 h post injection, analyzed using qPCR. **h**, **i** Expression levels of VP28 at mRNA (**h**) and protein (**i**) levels in hemocytes and intestine of *Ampkβ*-RNAi shrimp challenged by WSSV. **j**, **k** The relative expression levels of *IE1* (**j**) in hemocytes and intestine and the number of copies of WSSV (**k**) in intestine of the shrimp infected by WSSV, analyzed using qPCR. Significant differences were analyzed using Student’s *t* test, *P < 0.05; **P < 0.01; ***P < 0.001.

To explore the functions of AMPK in WSSV infection, RNA interference (RNAi) of *Ampkα* was performed and WSSV replication was analyzed using *Vp28* and *Ie1* expression as indicators. After successful knockdown of *Ampkα* at 36 h post *siAmpkα* injection (Fig. 2a, b, b′), WSSV replication was decreased significantly in hemocytes and intestines of the shrimp at the RNA (Fig. 2c) and protein levels (Fig. 2d) compared with that in the control group. Similar results were obtained for *Ie1* expression analysis (Fig. 2e). Meanwhile, the number of copies of WSSV also declined significantly in the hemocytes and intestines of *Ampkα*-knockdown shrimp (Fig. 2f). The above results indicated that knockdown of *Apmkα* expression reduced both WSSV gene expression and viral replication. To determine whether the three AMPK subunits have the same function in the process of WSSV infection, we interfered the *Ampkβ* expression by RNAi (Fig. 2g) and WSSV replication was analyzed in the *Ampkβ*-knockdown shrimp, and the same results were obtained (Fig. 2h–k). These findings suggest that activation of AMPK complex is beneficial for WSSV proliferation in vivo.

### Administration of an inhibitor or activator of AMPK confirms the viral beneficial function of AMPK in shrimp

**Fig. 3:**
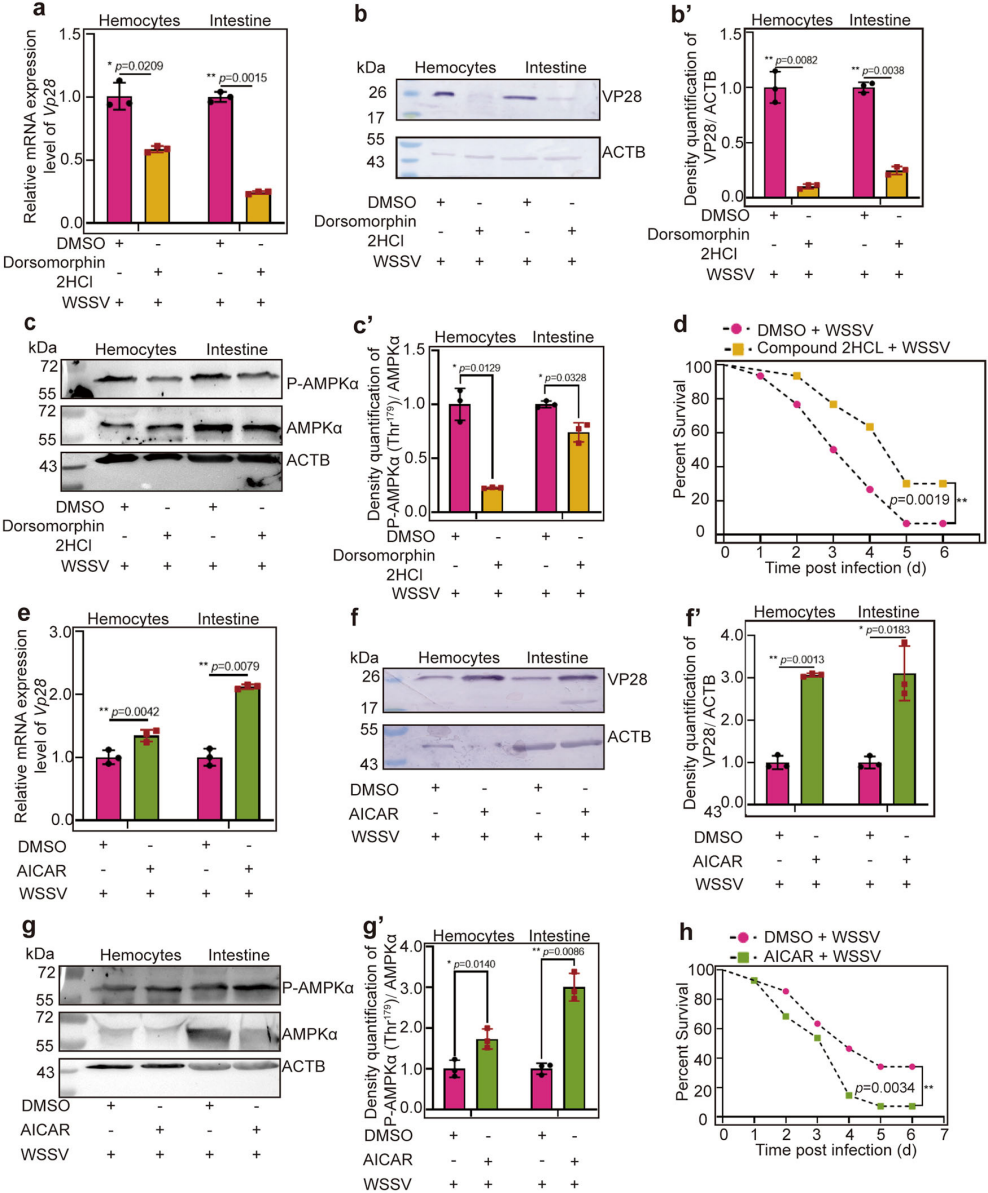
WSSV replication was decreased in inhibitor-injection shrimp and increased in activator-injection shrimp challenged by WSSV. **a**–**c** VP28 expression at the mRNA (**a**) and protein (**b**) levels and AMPK phosphorylation (**c**) in hemocytes and intestine of Dorsomorphin 2HCL-injection shrimp following WSSV infection, DMSO was used as control. **b′**, **c′** The statistical analysis of panel **b** or **c** based on three replicates. **d** Survival rate of the Compound 2HCL-injected shrimp challenged by WSSV (*n* = 30) and the control group injected with DMSO (*n* = 30). Significant differences were analyzed using the log-rank test in GraphPad Prism 8.0 software. **e**–**g** VP28 expression at the mRNA (**e**) and protein (**f**) levels and AMPK phosphorylation (**g**) in hemocytes and intestine of AICAR-injected shrimp following WSSV infection, DMSO was used as a control. **f′**, **g′** is the statistical analysis of panel **f** and **g** based on three replicates. **h** Survival rate of the AICAR-injected shrimp challenged by WSSV (*n* = 40) and the control group injected with DMSO (*n* = 40). Significant differences were analyzed using the log-rank test in GraphPad Prism 8.0 software. All results are shown as means ± SD for experiments performed at least three times; the data were analyzed statistically using Student’s *t* test. *P < 0.05; **P < 0.01; ***P < 0.001.

To confirm that activation of AMPK is beneficial for WSSV replication, an inhibitor (Dorsomorphin 2HCl) was injected into shrimp, and WSSV replication was analyzed. The toxic effect of Dorsomorphin 2HCl in shrimp was firstly detected by analyzing the survival rate of shrimp after Dorsomorphin 2HCl injection. The results showed that injection of Dorsomorphin 2HCl at different concentrations (2.5 μg, 5 μg, and 10 μg/g shrimp) did not reduce shrimp viability (Supplementary Fig. 4a). The effects of the inhibitor on WSSV proliferation in shrimp (using *Vp28* expression as the indicator) were analyzed using different concentrations. As shown in Supplementary Fig. 4b, c, Dorsomorphin 2HCl at 5 μg/g shrimp was used for subsequent study. After injection of Dorsomorphin 2HCl, VP28 expression at the mRNA (Fig. 3a) and protein (Fig. 3b, b′) levels and the level of phosphorylated AMPKα (Fig. 3c, c′) decreased significantly in hemocytes and intestines of the inhibitor injection group compared with those in the control. The survival rate of shrimp was also analyzed after inhibitor injection following WSSV infection. The results showed that the survival rates of the inhibitor Dorsomorphin 2HCL injection group increased significantly compared with that of the DMSO group (Fig. 3d).

Similarly, to confirm the function of AMPK, injection of AICAR, which can activate the kinase activity of AMPK^40,41^ was performed, and WSSV replication and AMPK phosphorylation were analyzed. First, the toxic effect of AICAR in shrimp was detected, and the results showed that the viability of shrimp was not affected at three different concentrations of AICAR (75 μg, 150 μg, and 250 μg/g shrimp) (Supplementary Fig. 4d). The effects of the activator on WSSV proliferation in shrimp were analyzed using different concentrations, and the results showed that WSSV proliferation was increased in a concentration dependent manner (Supplementary Fig. 4e, f). Therefore, AICAR at 150 μg/g shrimp was used in the following study. After injection of AICAR, WSSV replication and the level of phosphorylated AMPK in shrimp were detected. The results showed that VP28 expression at the mRNA (Fig. 3e), protein (Fig. 3f, f′) levels and the level of phosphorylated AMPK (Fig. 3g, g′) increased significantly in hemocytes and intestines of the AICAR injection group compared with those in the control. The survival rate of shrimp was also analyzed after activator injection following WSSV infection. The results showed that the survival rates of the activator AICAR injection group decreased significantly compared with that of the DMSO group (Fig. 3h). Taken together, these results suggested that AMPK was exploited by WSSV for its replication in shrimp.

### Ca^2+^/CaMKK signaling activated by WSSV infection is responsible for AMPK phosphorylation

**Fig. 4:**
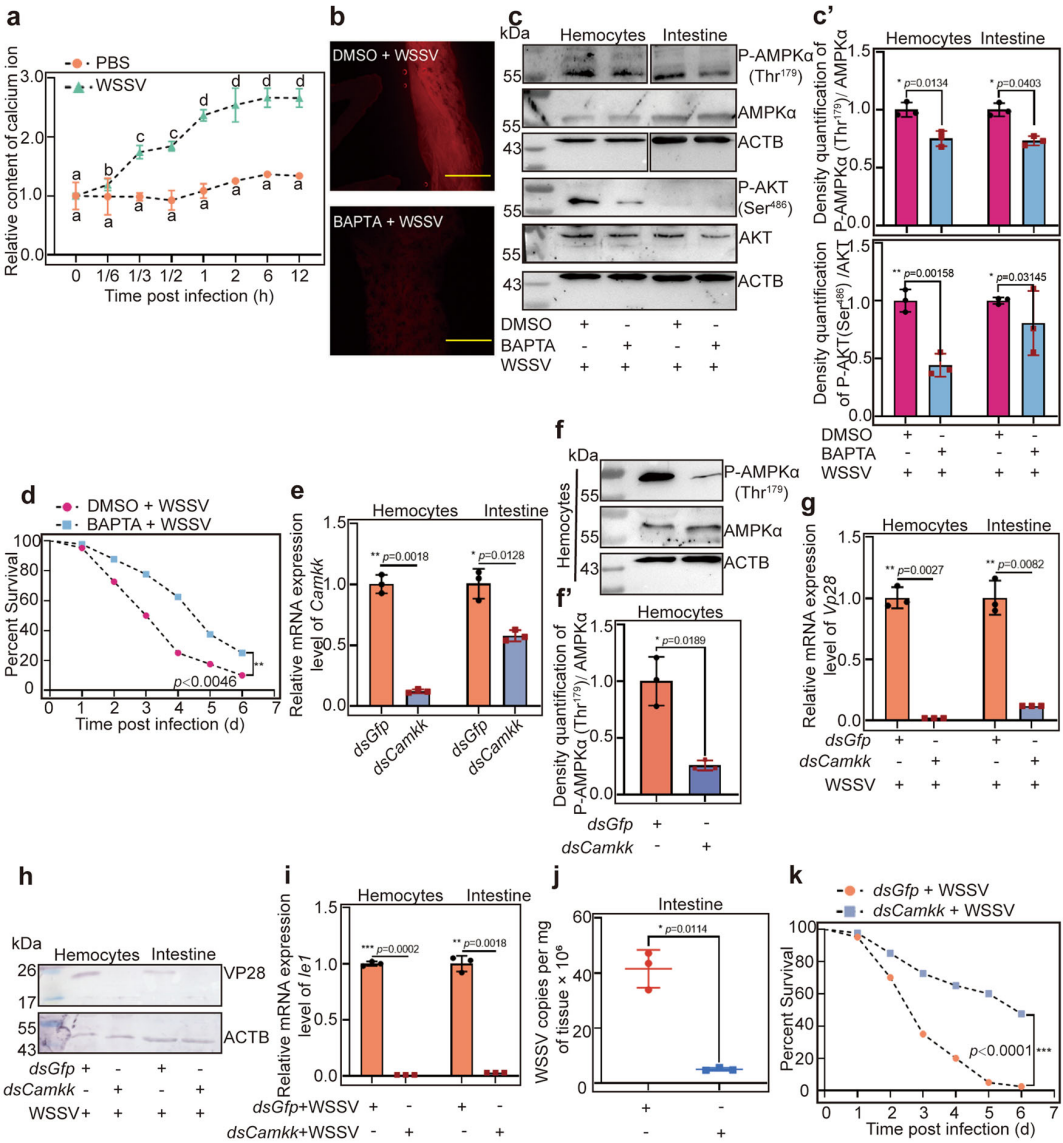
Ca^2+^ /CaMKK is responsible for phosphorylation of AMPK in shrimp challenged by WSSV. **a** Ca^2+^ concentration in the intestines of shrimp was detected at 0 (control), 10 min, 20 min, 30 min, 1, 2, 6, and 12 h post WSSV injection using a microplate reader. PBS injection was used as control. **b** Ca^2+^ concentration in the intestines of BAPTA-injected shrimp following WSSV infection was analyzed under a fluorescence microscope, DMSO injection following WSSV infection was used as the control group. Scale bar = 500 μm. **c** Phosphorylation levels of AMPK and AKT in BAPTA-injected shrimp following WSSV infection. Panel **c′** is the statistical analysis of panel **c** based on three replicates. **d** Survival rate of the BAPTA-injected shrimp challenged by WSSV (*n* = 40). DMSO injection was used as the control (*n* = 40). **e** The efficiency of *Camkk* interference in hemocytes and intestines, analyzed using qPCR. **f** Detection of phosphorylated AMPKα (p-AMPKα) in hemocytes of *Camkk*-RNAi shrimp following WSSV infection, analyzed using western blotting. **f′** is the statistical analysis of panel **f** based on three replicates. **g**, **h** VP28 expression at the mRNA (**g**) and protein (**h**) levels in hemocytes and intestine of *Camkk*-knockdown shrimp following WSSV challenge, analyzed using qPCR and western blotting. **i**, **j** *Ie1* expression (**i**) and viral copy number (**j**) of WSSV in the *Camkk*-knockdown shrimp. **k** Survival rate of the *Camkk*-knockdown shrimp challenged by WSSV (*n* = 40) and *dsGfp* injection was used as the control (*n* = 40). Significant differences were analyzed using the log-rank test in GraphPad Prism 8.0 software. All results are shown as means ± SD for experiments repeated at least three times; the data were analyzed statistically using Student’s *t* test. *P < 0.05; **P < 0.01; ***P < 0.001.

Studies have shown that Ca^2+^/CaMKK signaling can activate AMPK^32,42^. To determine whether WSSV infection affects the level of Ca^2+^, the free cytosol Ca^2+^ in intestine of shrimp was detected using a Calcium Colorimetric Assay. The results showed that the intracellular calcium concentration was increased by WSSV infection in shrimp compared with that in the control group injected with PBS (Fig. 4a). To further verify that WSSV can activate AMPK through the calcium signaling pathway, we injected the Ca^2+^ chelator BAPTA and observed phosphorylation of AMPK. The results showed that the Ca^2+^ level was reduced significantly (Fig. 4b) and the level of phosphorylated AMPK decreased significantly in the BAPTA-injection group following WSSV infection compared with that in the control group (Fig. 4c, c′ upper panel). The phosphorylation of AKT, the downstream protein of AMPK, also decreased significantly in the BAPTA-injection group (Fig. 4c, c′ lower panel). The survival rates of the calcium chelator BAPTA injection group increased significantly compared with that of the DMSO group (Fig. 4d).

Previous reports have shown that CaMKK (GenBank accession no. XP042859763) regulates AMPK phosphorylation in mammals^43^. To analyze whether CaMKK is involved in the AMPK signaling pathway in *M. japonicus*, RNAi was used to knock down *Camkk* and AMPKα phosphorylation and WSSV replication were detected in shrimp. The results showed that after knockdown of *Camkk* (Fig. 4e), AMPK phosphorylation (Fig. 4f, f′), VP28 mRNA (Fig. 4g), and protein (Fig. 4h) levels, as well as *Ie1* (Fig. 4i) expression levels were decreased significantly in hemocytes and intestine of *Camkk*-RNAi shrimp following WSSV challenge. The viral copy number also declined significantly (Fig. 4j). Furthermore, the survival rates of the *dsCamkk*-injection group following WSSV injection increased significantly compared with that of the *dsGfp* group (Fig. 4k). These results suggested that as an upstream kinase of AMPK, Ca^2+^-activated CaMKK phosphorylates AMPK to promote WSSV replication.

### WSSV infection induces partially nuclear translocation of AMPKα

**Fig. 5:**
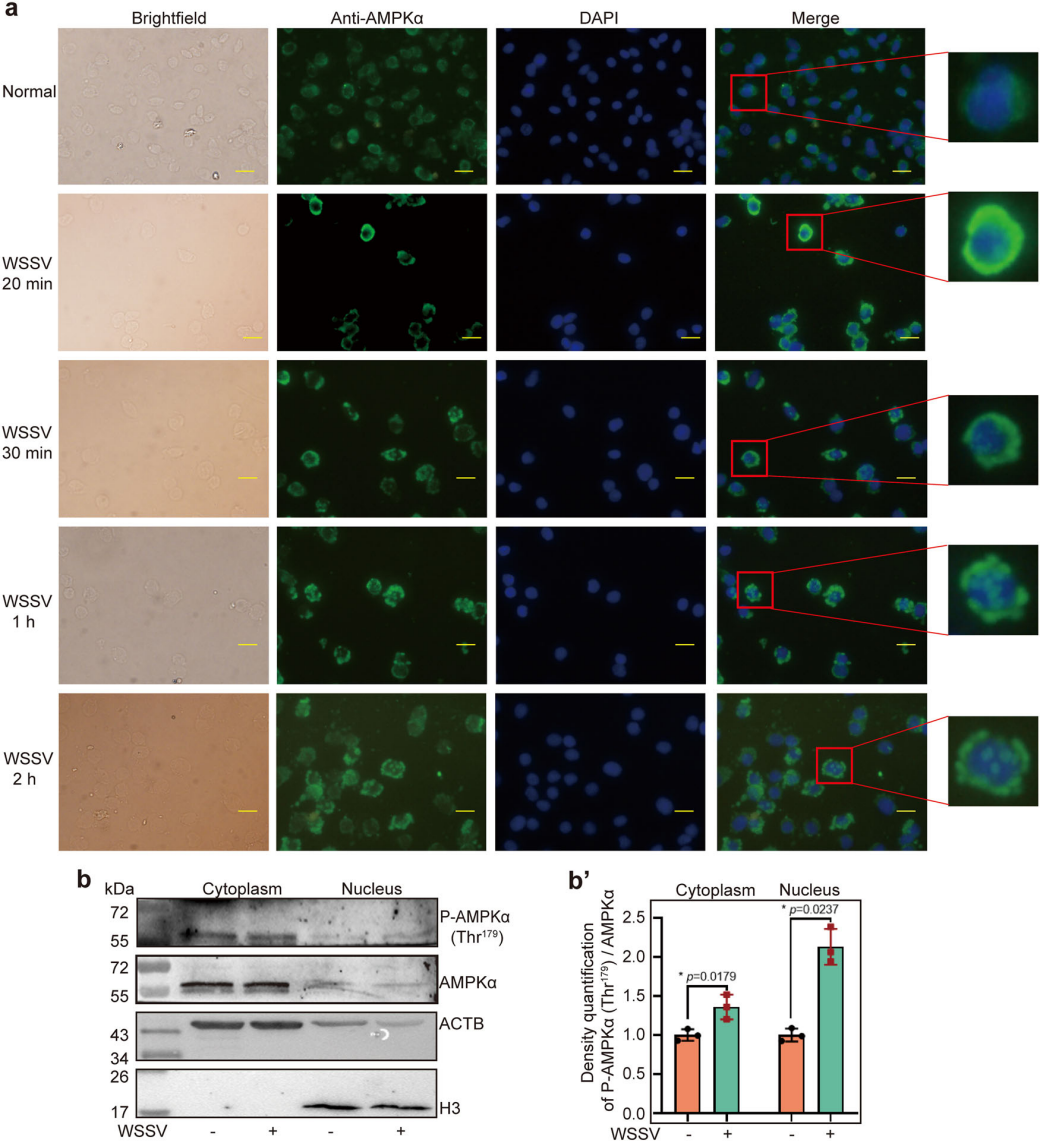
AMPK is translocated into the nucleus partially in hemocytes and intestine of shrimp challenged by WSSV. **a** The nuclear translocation of AMPK in hemocytes was detected at 0, 20 min, 30 min, 1 h, and 2 h post WSSV challenge using a fluorescent immunocytochemical assay. Scale bar = 10 μm. **b** Nuclear translocation of AMPKα in the intestines analyzed using western blotting, **b′** The statistical analysis of panel b based on three replicates.

Cell compartmentalization of AMPK is closely related to its function. We performed a fluorescent immunocytochemical assay to detect the dynamic subcellular distribution of AMPKα in hemocytes during WSSV infection. The results showed that that AMPKα was distributed mainly in the cytosol of hemocytes in untreated shrimp and started to partially translocate into the nucleus at 20 min post WSSV injection. In addition, the AMPK signal could be detected in most cell nuclei of hemocytes in addition to cytosol at 2 h post WSSV injection (Fig 5a). Similar results were obtained in intestine cells (Fig 5b, b′) analyzed by western blot. The data suggested that AMPK partially translocated into nucleus and functions in the cytosol and nucleus of hemocytes in shrimp challenged by WSSV.

### AMPK plays a beneficial role in viral replication via mTORC2-AKT signaling

**Fig. 6:**
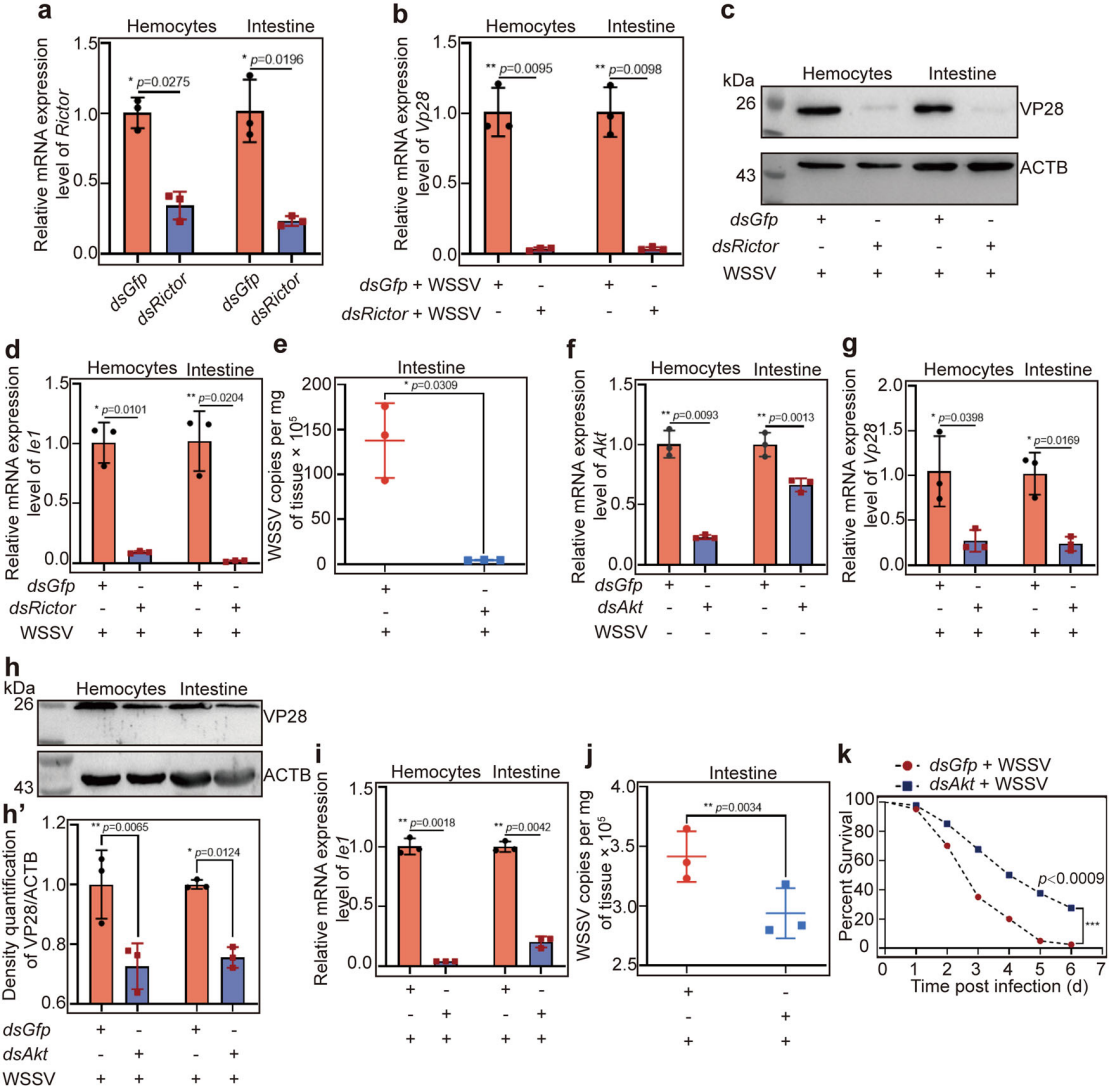
mTORC2-AKT signaling activation promotes WSSV replication. **a** The efficiency of *Rictor* interference assays in hemocytes and intestines. **b**, **c** *Vp28* expression at the mRNA (**b**) and protein (**c**) levels in hemocytes and intestine of *Rictor*-knockdown shrimp following WSSV challenge, analyzed using qPCR and western blotting. **d**, **e** *Ie1* expression (**d**) and copy number (**e**) of WSSV in the shrimp. **f** The efficiency of *Akt* RNAi in hemocytes and intestines. **g**, **h** *Vp28* expression at the mRNA (**g**) and protein (**h**) levels in hemocytes and intestine of Akt-knockdown shrimp following WSSV challenge, analyzed using qPCR and western blotting. **h′** is the statistical analysis of panel **h** based on three replicates. **I**, **j** *Ie1* expression (**i**) and copy number (**j**) of WSSV in the shrimp. **k** Survival rate of the *dsAkt*-injection shrimp challenged by WSSV (*n* = 40), *dsGfp* injection shrimp challenged by WSSV was used as the control group (*n* = 40). Significant differences were analyzed using the log-rank test in GraphPad Prism 8.0 software. All results are shown by means ± SD for experiments performed at least three times; the data were analyzed statistically using Student’s *t* test. *P < 0.05; **P < 0.01; ***P < 0.001.

It was unexpected that AMPK was beneficial for WSSV replication in shrimp, because AMPK has traditionally been thought to inhibit mTORC1 signaling, and several studies found that WSSV utilizes the PI3K-Akt-mTORC1 pathway for its proliferation in shrimp^25^. A recent study found that AMPK directly activated mTORC2-AKT signaling and promoted cell survival in response to energetic stress in mammalian HEK293 cells^24^. mTORC2 phosphorylates AKT (Ser^473^) at its hydrophobic motif site^44^ and promotes metabolism^45^. Therefore, we hypothesized that activation of AMPK by WSSV infection could directly activate mTORC2-AKT signaling to promote WSSV proliferation. We found that the expression of *Rictor* (GenBank no. OK143319), the core component of mTORC2 was upregulated in shrimp challenged by WSSV (Supplementary Fig. 5). We then performed RNAi to knock down *Rictor* and analyzed WSSV replication. The results showed that after knockdown of *Rictor* (Fig. 6a), the expression of VP28 (Fig. 6b, c) and *Ie1* (Fig. 6d), and the virus copy number (Fig. 6e) decreased significantly in the hemocytes and intestines of shrimp challenged by WSSV. We further performed RNAi to knock down *Akt* (KP419299), the downstream molecule of mTORC2 (Fig. 6f), and same results were obtained for VP28 expression (Fig. 6g, h, h′), *Ie1* expression (Fig. 6i), and viral copies (Fig. 6j). The survival rate of shrimp was also analyzed after *dsAkt* injection following WSSV infection. The results showed that the survival rates of the *Akt*-knockdown group increased significantly compared with that of the *dsGfp* group (Fig. 6k). mTORC2, AKT, and AMPK have the same effect in promoting WSSV replication in the host. Therefore, all the results suggested that WSSV infection activated AMPK, and then induced activation of mTORC2-AKT signaling to promote WSSV proliferation.

### AMPK directly activates mTORC2-AKT signaling

**Fig. 7:**
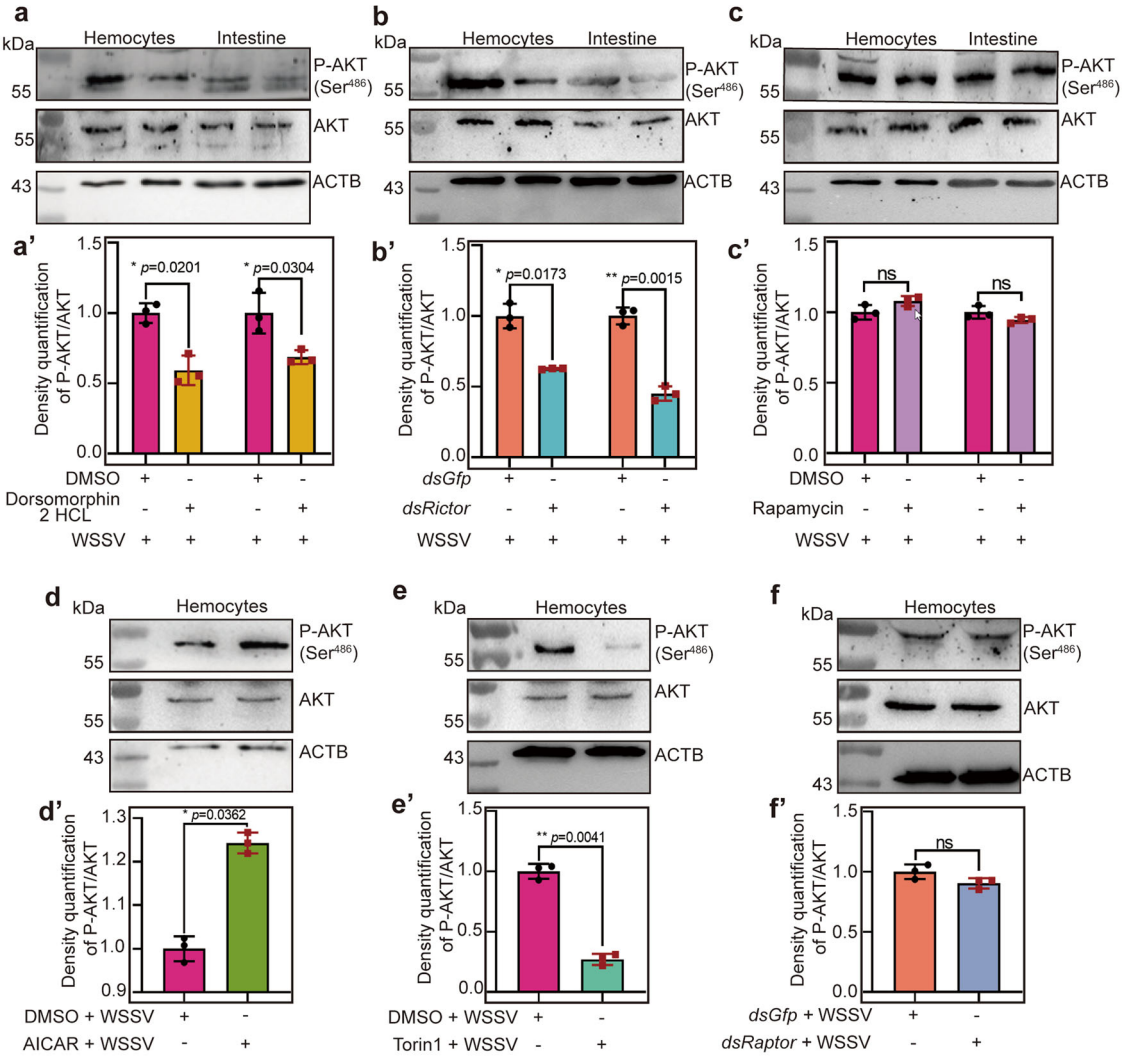
AMPK activates the mTORC2-AKT pathway directly. **a** AKT phosphorylation analysis at 2 h post Dorsomorphin 2HCl injection in shrimp following WSSV infection, using western blotting with human p-AKT (Ser^473^) antibodies. Shrimp injected with the same amount of DMSO were used as controls. **a′** The statistical analysis of panel **a** based on three replicates. **b** AKT phosphorylation analysis in *Rictor*-knockdown shrimp following WSSV infection, using western blotting with human p-AKT (Ser^473^) antibodies. *dsGfp* injection was used as a control. **b′** The statistical analysis of panel **b** based on three replicates. **c** AKT phosphorylation analysis at 2 h post Rapamycin injection in shrimp following WSSV infection, using western blotting with human p-AKT (Ser^473^) antibody. Shrimp injected with the same amount of DMSO were used as controls. **c′** The statistical analysis of panel **c** based on three replicates. **d** AKT phosphorylation analysis at 2 h post AICAR injection in shrimp following WSSV infection, using western blotting with human p-AKT (Ser^473^) antibodies. Shrimp injected with the same amount of DMSO used as controls. **d′** The statistical analysis of panel **d** based on three replicates. **e** AKT phosphorylation analysis at 2 h post Torin1 injection in shrimp following WSSV infection, using western blotting with human p-AKT (Ser^473^) antibodies. Shrimp injected with the same amount of DMSO used as controls. **e′** The statistical analysis of panel **e** based on three replicates. **f** AKT phosphorylation analysis in *Raptor*-knockdown shrimp following WSSV infection, using western blotting with human p-AKT (Ser^473^) antibodies. Shrimp injected with the same amount of *dsGfp* as controls. **f′** The statistical analysis of panel **f** based on three replicates. ACTB was used as loading control in western blotting. All results are shown by means ± SD for experiments were performed at least three times; the data were analyzed statistically using Student’s *t* test. *P < 0.05; **P < 0.01; ***P < 0.001.

To further confirm that AMPK can directly activate mTORC2-AKT signaling to promote viral proliferation in shrimp infected by WSSV, we injected Dorsomorphin 2HCl or AICAR into shrimp to observe changes of the level of phosphorylated AKT after WSSV infection using a human anti-p-AKT (Ser^473^) antibody (the phosphorylation site of shrimp AKT is Ser^486^) (Supplementary Fig. 6), which is a hallmark of mTORC2 activation^46^. After the injection of the inhibitor, Dorsomorphin 2HCl, the level of phosphorylated AKT decreased significantly compared with that in the control group (Fig. 7a, a′). We also found that knockdown of *Rictor* reduced the level of phosphorylated AKT in shrimp significantly (Fig. 7b, b′). In addition, we observed no effect on the level of phosphorylated AKT after the injection rapamycin, the inhibitor of mTORC1 (Fig. 7c, c′).

To further verify our results, an activator of AMPK, AICAR, and inhibitor of mTORC1/2, Torin1, were injected into shrimp, separately, and AKT phosphorylation was analyzed. We found that the level of phosphorylated AKT increased significantly in the hemocytes after injection of AICAR (Fig. 7d, d′). Moreover, after injection of Torin1, phosphorylation of AKT was impaired in the hemocytes of shrimp challenged by WSSV (Fig. 7e, e′). In addition, after knockdown of *Rapt*or (Regulatory-associated protein of mTOR, XP_042857230.1), the core component of mTORC1, the level of phosphorylated AKT was not affected (Fig. 7f, f′). All the results suggested that activation AMPK by WSSV infection directly activates the mTORC2 signaling pathway to promote WSSV replication.

### AMPK induced mTORC2-AKT signaling to promote the glycolytic pathway in shrimp infected by WSSV

**Fig. 8:**
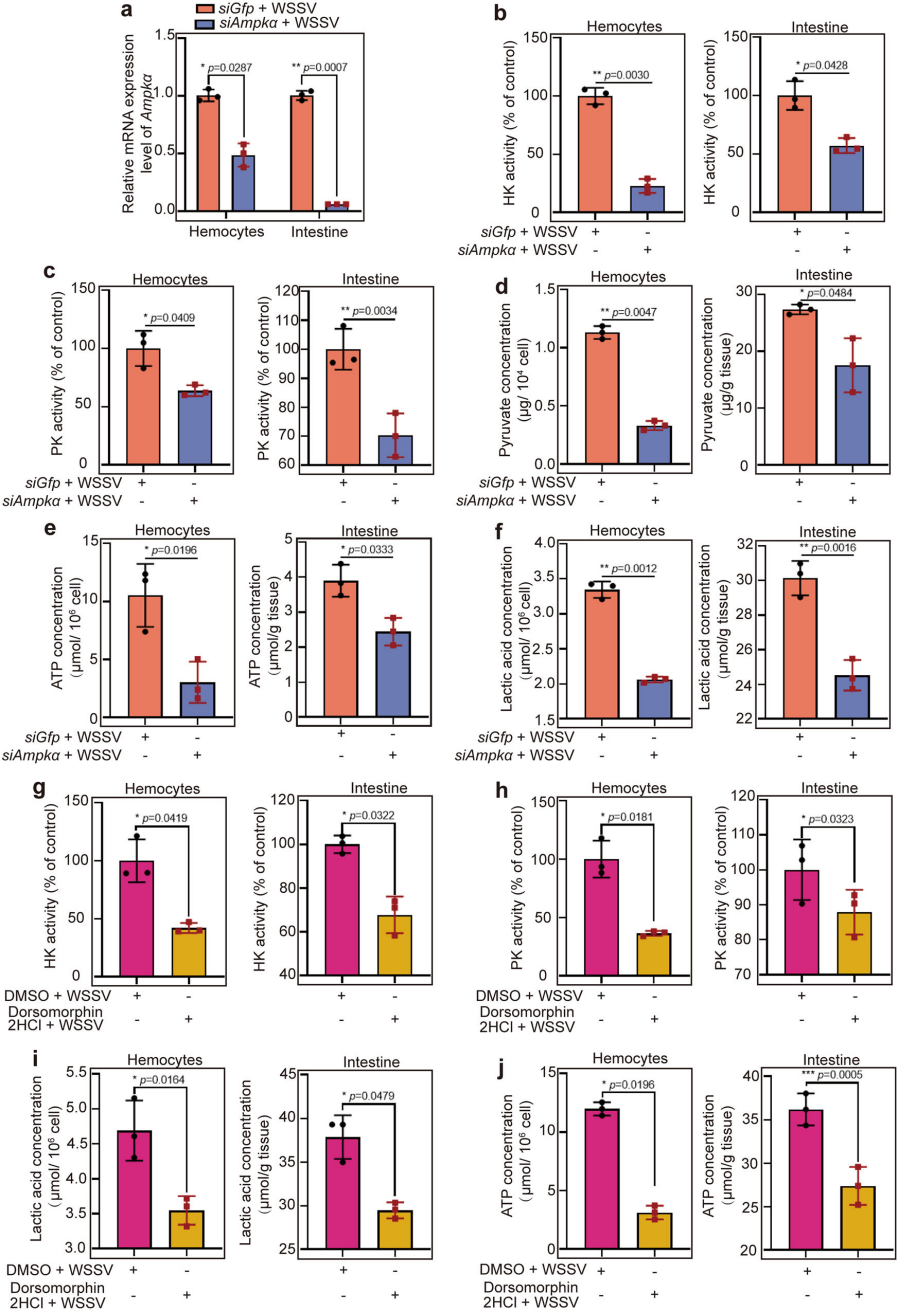
Knockdown or activity inhibition of AMPK impaired the glycolytic pathway in shrimp induced by WSSV infection. **a** The efficiency of *Ampkα* interference in hemocytes and intestines. **b**, **c** The enzyme activity of HK (**b**) and PK (**c**) in hemocytes and intestine of *Ampk-*knockdown shrimp challenged by WSSV. **d**-**f** The contents of pyruvate (**d**), ATP (**e**), and lactic acid (**f**) in hemocytes and intestine of *Ampk*-knockdown shrimp challenged by WSSV. **g**, **h** The enzyme activity of HK (**g**) and PK (**h**) in hemocytes and intestine of Dorsomorphin 2HCl-injection shrimp following WSSV infection; and DMSO was used as a control. **i**, **j** The contents of lactic acid (**i**), and ATP (**j**) in hemocytes and intestine of Dorsomorphin 2HCl-injection shrimp following WSSV infection; DMSO was used as a control. All results are shown as means ± SD for experiments performed at least three times; the data were analyzed statistically using Student’s *t* test. *P < 0.05; **P < 0.01; ***P < 0.001.

AMPK is a central energy metabolism regulator that activates glycolysis through multiple steps in the glycolysis pathway, including increased glucose uptake and phosphofructokinase activity^47^. To analyze AMPK’s involvement in glycolysis via mTORC2-AKT signaling in WSSV-infected shrimp, we detected the activity of key enzymes [hexokinase (HK) and pyruvate kinase (PK)] in glycolysis and the contents of glycolytic metabolites, including pyruvic acid (PA), ATP, lactic acid (LA) in the *Ampka*-RNAi shrimp. After knockdown of *Ampkα* (Fig. 8a) in shrimp, followed by WSSV infection, the enzyme activity of HK (Fig. 8b) and PK (Fig. 8c) decreased significantly in hemocytes and intestines. The contents of glycolytic metabolites PA (Fig. 8d) and ATP (Fig. 8e) also decreased significantly. At the same time, we detected the content of LA (Fig. 8f), which was decreased significantly compared with that in the control, suggesting that aerobic glycolysis occurred in WSSV-infected shrimp. However, during WSSV infection in shrimp, the enzyme activity of HK (Supplementary Fig. 7a) and PK (Supplementary Fig. 7b) increased significantly in hemocytes and intestines. Similarly, levels of glycolytic metabolites PA (Supplementary Fig. 7c), ATP (Supplementary Fig. 7d), and LA (Supplementary Fig. 7e) also increased. These results suggested that knockdown of *Ampk* impaired the glycolysis pathway, thus providing less energy for WSSV replication.

To further confirm that activated AMPK enhances glycolysis via mTORC2-AKT signaling, we injected a Dorsomorphin 2HCl into shrimp, followed by WSSV infection, and detected key enzyme activity and metabolite contents. Similar to the results of RNAi, inhibition of AMPK activity reduced the activity of the key enzymes HK (Fig. 8g) and PK (Fig. 8h) in glycolysis and the contents of glycolytic metabolites, lactic acid (Fig. 8i), and ATP (Fig. 8j). Taken together, these results suggested that AMPK activates the mTORC2-AKT axis resulting in enhanced glycolysis to provide energy for WSSV replication.

### AMPK promotes expression of hypoxia-inducible factor 1 to mediate the expression of glycolytic genes

**Fig. 9:**
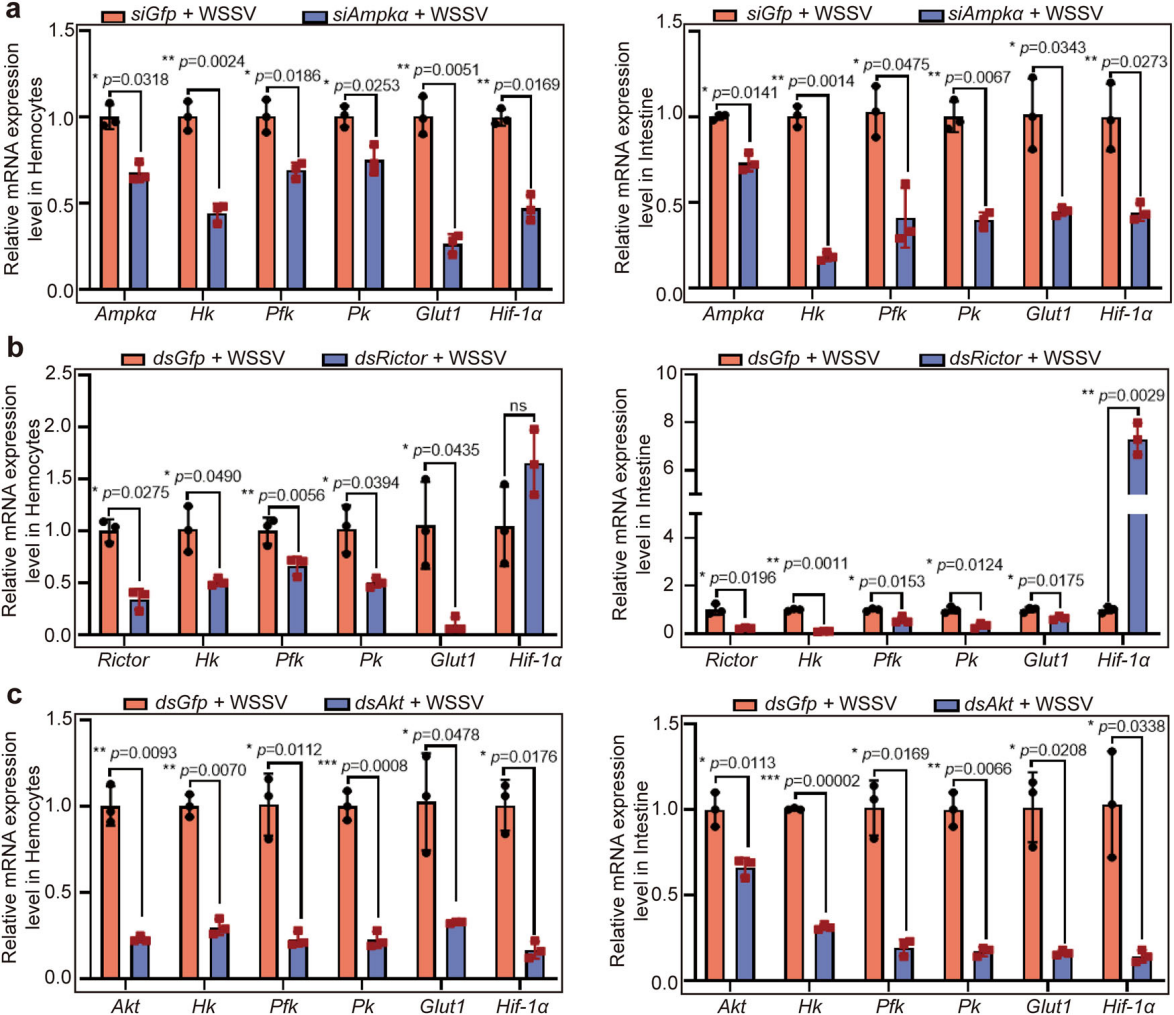
The mRNA expression levels of glycolysis-related genes declined after knockdown of *Ampkα*, *Rictor*, and *Akt* in shrimp challenged by WSSV. **a** Efficiency of *Ampkα* RNAi and the mRNA expression levels of *Hk*, *Pfk*, and *Pk* in hemocytes and intestine in the shrimp analyzed by qPCR. **b** Efficiency of *Rictor* RNAi, and the mRNA expression levels of *Hk*, *Pfk*, and *Pk* in hemocytes and intestine in the shrimp analyzed by qPCR. **c** Efficiency of *Akt* RNAi, and the mRNA expression levels of *Hk*, *Pfk*, and *Pk* in hemocytes and intestine in the shrimp analyzed by qPCR. All results shown are as means ± SD for experiments performed at least three times; the data were analyzed statistically using Student’s *t* test. *P < 0.05; **P < 0.01; ***P < 0.001.

We found that WSSV infection induced partial nucleus translocation of AMPKα (Fig. 5). A previous report indicated that HIF-1 regulates WSSV-induced glycolytic genes in shrimp^48^. To further reveal the link between AMPK and glycolytic gene expression in WSSV-infected shrimp, we knocked down *Ampka* and analyzed expression of HIF1α (XP_042868035.1), hexokinase (HK, OL364939), phosphofructokinase (PFK, OL364940), and pyruvate kinase (PK, OL364941) and glucose transporter (Glut1, OL364942) at the RNA level. The results showed that the mRNA expression level of *Hif1α* was decreased significantly in hemocytes and intestines; meanwhile, the expression levels of *Hk*, *Pfk*, *Pk*, and *Glut1* were also decreased in hemocytes and intestines in the shrimp challenged by WSSV (Fig. 9a). After knockdown of *Rictor* (OK143319) in shrimp infected by WSSV, the expression of the key enzyme genes in glycolysis, including *Glut1*, *Hk*, *Pfk*, *and Pk*, decreased significantly in hemocytes and intestines (Fig. 9b). Interestingly, knocking down *Rictor* did not affect the expression of *Hif1α*, suggesting that mTORC2 could not regulate *Hif1α* expression. After knockdown of *Akt* in WSSV-infected shrimp, the expression of glycolytic enzyme genes decreased significantly, as expected, surprisingly, the expression of *Hif1α* also decreased (Fig. 9c). The reason might be that AKT also regulates *Hif1α* expression through other signaling pathways^49,50^. Several downstream targets of AKT, such as NF-κB, were reported, therefore, we detected the expression of NF-κB like transcription factors, Dorsal (AME17867) and Relish (QPB70448), in the *Akt*-knockdown shrimp. The results showed that transcripts of *Dorsal* and *Relish* were significantly increased in intestine in AKT knockdown shrimp after WSSV infection, but no significant change in hemocytes (Supplementary Figure 8). To confirm the expression of glycolytic genes was regulated by HIF1α, the in vitro binding activity of rHIF1α to the binding site in the promoter region of HK was detected by electrophoretic mobility shift assay (EMSA). The result showed that rHIF1α interacted with binding site of HK promoter. And the GST control protein could not bind to HK probes (Supplementary Fig. 9). These results suggested that activated AMPK might positively regulated transcription factor *Hif1α* in an indirect way to promote the expression of glycolytic enzyme genes.

## Discussion

**Fig. 10:**
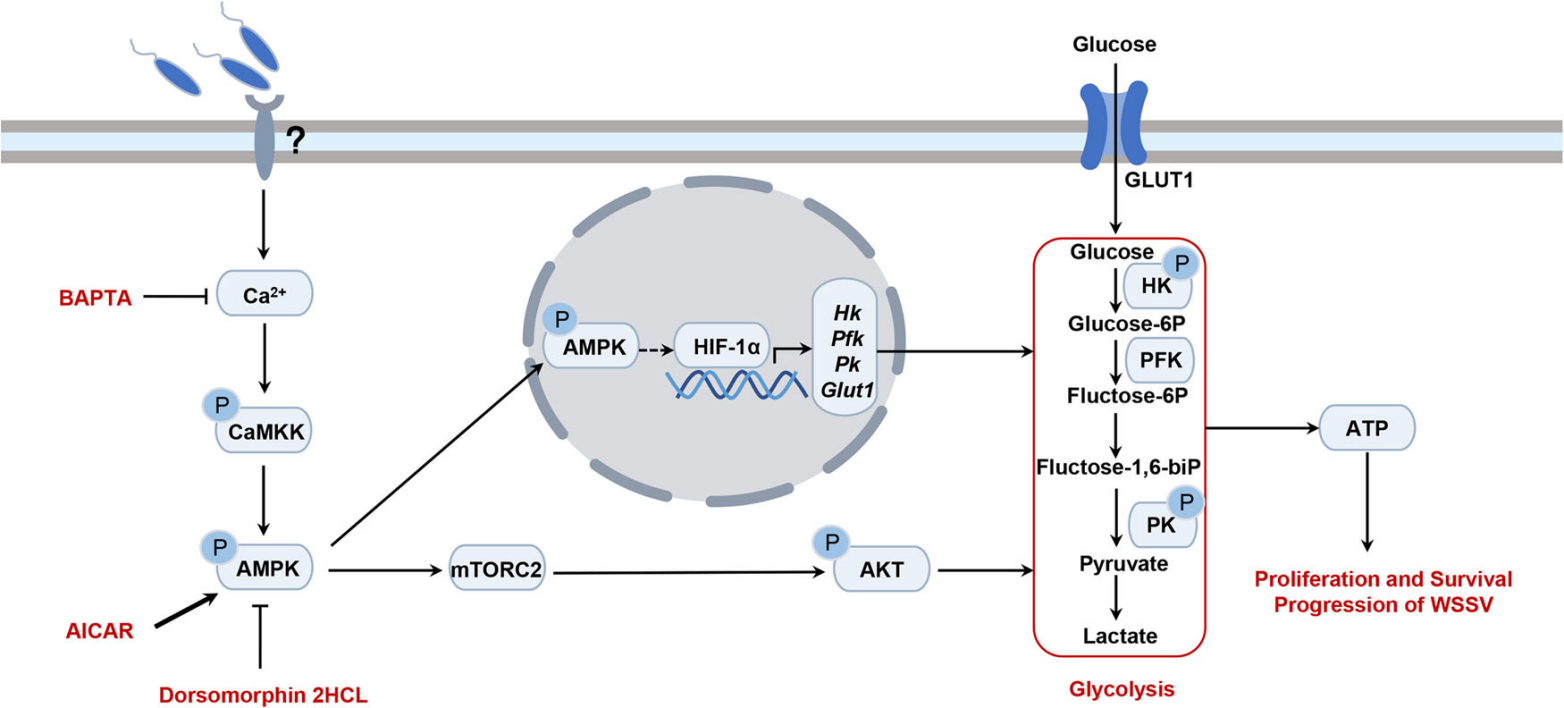
Schematic representation of AMPK activating mTORC2 signaling to promote viral proliferation. WSSV activates AMPK through the Ca^2+^-CAMKK signaling pathway, and AMPK promotes mTORC2-AKT signaling to positively modulate the glycolysis pathway to provide energy for replication of WSSV in the host. In addition, AMPK can enter the nucleus to activate transcription factors and positively regulate glycolysis-related gene expression.

In the present study, we investigated the role of AMPK in *M. japonicus* infected by WSSV, and found that WSSV infection induces intracellular Ca^2+^ release, resulting in CaMKK activation that phosphorylates AMPK and induces its partial nuclear translocation. AMPK in the cytosol directly activates the mTORC2-AKT pathway to enhance glycolysis for energy production used for WSSV propagation. AMPK in the nucleus induces the expression of *Hif1α* to positively regulate the expression of glycolytic enzyme genes (Fig. 10). All the data suggest that WSSV utilizes AMPK to promote glycolysis to increase the energy supply for viral proliferation via the mTORC2-AKT axis in cell cytosol and by promoting HIF1α expression for transcription of key enzyme genes of glycolysis in the shrimp cell nucleus. This is the first systematic study of the function of AMPK in viral infection.

Host cells modulate calcium-signaling components on cell plasma membrane in response to viral infection. On the other hand, viruses utilize these components to create a cellular environment that benefits viral lifecycles. Ca^2+^ is essential for virus entry, viral gene replication, virion maturation, and release^51^. The alteration of host cells Ca^2+^ homeostasis is one of the strategies that viruses use to modulate host cells signal transduction mechanisms during viral infection (see the reviews by refs. ^51,52^). For example, influenza A virus, HIV-1 and herpes simplex virus (HSV)-1 can control host voltage-gated calcium channels (VGCCs) beneficial for viral replication^51,53^; Hepatitis B virus (HBV) can also elevate cytosolic Ca^2+^ levels, the activation of intracellular Ca^2+^ signaling contributes to viral replication via multiple molecular mechanisms within HBV-infected cells^54^. In our study, we found that the intracellular Ca^2+^ increased significantly in cells of shrimp infected by WSSV, and this might be related with activation of CaMKK and AMPK in shrimp.

There are at least three pathways that result in AMPK activation, including adenine nucleotide-dependent, Ca^2+^-dependent, and D-fructose-1,6-bisphosphate (FBP)-dependent pathways^55,56^. Previous reports also found that AMPK activation is confined to the cytosol in response to energy stress^56^, whereas the activation of AMPK can be observed in both the cytosol and nucleus in response to increased intracellular Ca^2+^^57^. Several studies have identified CaMKKβ as the upstream kinases that phosphorylates Thr-172 in the activation loop of AMPKα^58^. There are distinct subcellular pools of AMPK, including lysosomal-associated AMPK, cytosolic AMPK, mitochondrial-associated AMPK, and nuclear-associated AMPK in mammalian cells^55,56^. The activation of AMPK in the nucleus leads to changes in gene expression by phosphorylation of transcription factors^55^. In the present study, we found that AMPK was mainly distributed in the cytosol of hemocytes, and was partially translocated into nucleus during WSSV infection. In the cytosol, WSSV infection increased the Ca^2+^ level, and CaMKK was activated by the elevated calcium. As an upstream kinase, CaMKK induced AMPK activation in Ca^2+^-dependent manner. Similar to mammals, in shrimp, AMPK directly activated mTORC2-AKT signaling and induced glycolysis by phosphorylating key enzymes of the pathway. Meanwhile, activated AMPK in the nucleus phosphorylated transcription factors, such as HIF-1α, to increase the expression of glycolysis-related genes.

AMPK has multifaceted roles in viral infection^12^. AMPK decreases HBV replication through the promotion of autolysosome-dependent degradation^13^. Activation of AMPK restricts Coxsackie virus B3 (CVB3) replication by the inhibition of lipid accumulation^15^. On the other hand, CVB3 induces autophagy via AMPK/MEK/ERK and Ras/Raf/MEK/ERK signaling pathways, which are essential for the life cycle of CVB3^59^. WSSV hijacks the host metabolome via the PI3K-AKT-mTOR pathway to achieve successful replication in shrimp^25^. In the present study, we identified a novel strategy, in which WSSV infection in shrimp hijacks the Ca^2+^/CaMKK-AMPK-mTORC2-AKT axis and Ca^2+^/CaMKK-AMPK-HIF1α axis to promote viral proliferation.

WSSV infection induces host several metabolic reprogramming, such as glycolysis, the tricarboxylic acid (TCA) cycle, glutaminolysis and lipid metabolism, for viral survival and replication^60^. Aerobic glycolysis is one of the major pathways that provide energy for viral replication. The metabolisms are regulated by different signal pathways, such as The PI3K-AKT-mTOR pathway^25^, PI3K/AKT signaling pathway^61^ and MAPK-ERK-MEK pathway^60^. WSSV infection increases glucose consumption and lactate accumulation (Warburg effect)^62^. The PI3K-Akt-mTOR signaling pathway is involved in triggering the WSSV-induced Warburg effect, and that its activation is beneficial to WSSV replication. In shrimp hemocytes, the mTORC1 pathway is predominant for WSSV gene expression, while inhibition of mTORC1 alone is not sufficient to reduce WSSV replication and the mTORC2 pathway might be involved in triggering the Warburg-like effect^25^. The mTORC2 is reported as a critical regulator of cancer cell metabolism through two mechanisms: Akt-dependent and Akt-independent signaling, leads to the acetylation of FoxO1 and FoxO3, causing release of c-Myc from a suppressive microRNA network^63^. How the mTORC2 involved in Warburg-like effect in shrimp is not clear. Here, we found that phosphorylated AMPK activates the mTORC2-AKT axis to promote aerobic glycolysis for energy production used in WSSV propagation in the host cells.

AKT is also reported involving in different signaling pathways in addition to energy metabolism. Activated AKT can lead to inhibition of caspase-9 activity, or initiation of nuclear factor-κB (NF-κB) activation^64^. In shrimp, we also found that transcripts of the NF-κB like transcription factors, Dorsal and Relish were significantly increased in the *Akt*-knockdown shrimp after WSSV infection, suggesting that the NF-κB pathway was inhibited by the mTORC-AKT axis. The NF-κB pathways, including Toll and IMD pathways, play important roles in antiviral immunity in shrimp by regulating expression of antimicrobial peptides^65^. Therefore, our results suggested that AMPK-mTORC2-AKT axis might be involved in the inhibition of shrimp NF-κB pathways.

The transcription factor HIF1α is not only regulated by hypoxia, but also in response to various stresses, hormones, or cytokines^66^. Phosphorylation of HIF1α was necessary for its transcriptional activity^67^. Several different kinases have been identified to regulate HIFα phosphorylation in a direct or indirect fashion^67^. For example, a study in *Caenorhabditis elegans* indicated that AMPK phosphorylates HIF-1α in a direct manner^68^. The regulation of HIF-1α by AMPK also reported in the human hepatic cancer cell^69^. The study demonstrates that AMPK signaling regulates the nuclear accumulation of HIF-1α protein in indirect way. AMPK phosphorylates histone deacetylase 5 (HDAC5), and induces cytosolic shuttling of HDAC5, which is a crucial step in the proper accumulation of HIF-1α protein into nuclei and subsequent induction of hypoxia response. In our study, we found that AMPK was partially translocated into nucleus after WSSV infection, and the Hif1α was downregulated significantly after knockdown of AMPK, suggesting AMPK promoting expression of Hif1α in an unknow fashion, and mediating the expression of glycolytic genes. The detail mechanism needs further study.

PK is a key enzyme that catalyzes the conversion of phosphoenolpyruvate (PEP) to pyruvate in the glycolysis pathway. Some reports showed that PK activity decreased after WSSV infection^26^, whereas other studies found that PK activity increased after WSSV infection^48^. In addition, WSSV infection increases the protein level of PK in hemocytes of WSSV infected shrimp. Our results showed that PK activity and other enzymes, such as HK and PFK, increased in shrimp after WSSV infection, which suggests an increase in the flow of glycolysis to provide energy for the viral proliferation.

In conclusion, we found that WSSV infection activates AMPK through the Ca^2+^-CAMKK cascade. Activated AMPK induces the activation of mTORC2-AKT axis, and AKT, as a central regulator of glucose metabolism, upregulates activity of glycolytic enzymes to promote glycolysis to produce energy, which is beneficial to WSSV proliferation in shrimp. Alternatively, AMPK can enter the nucleus to promote expression of the transcription factor, HIF1α, in an unknown fashion and promote expression of glycolytic enzyme genes. Our data suggest that AMPK could be a novel target for WSSV control in the shrimp industry.

## Methods

### Animals

Shrimp *M. japonicus* (~6-12 g each) were purchased from a breeding farm in Qingdao, Shandong Province, China. The shrimp were cultured in the laboratory for more than 2 days in aerated seawater at ~24 °C for acclimation and then randomly selected for subsequent experiments.

### Phylogenetic and sequence analysis

The cDNA sequences encoding AMPKα, β and γ were obtained from hemocyte transcriptome sequencing of *M. japonicus*. Sequence similarity analysis was performed using BLAST (http://www.ncbi.nlm.nih.gov/). The deduced amino acid sequences, molecular weight calculation, and isoelectric point analysis were carried out using ExPASy (https://web.expasy.org/). Protein domain prediction was performed using SMART (http://smart.embl-heidelberg.de/index2.cgi). Sequence alignments were executed using Genedoc (http://www.psc.edu/biomed/genedoc), and MEGA 5.05 (http://www.megasoftware.net) was used to construct phylogenetic trees of AMPKs from different species.

### RNA and protein extraction, and cDNA synthesis

Total RNA was extracted from the hemocytes and other organs of shrimp using the TRIzol (ET101, Transgen, Beijing, China). Proteins from the different organs were extracted using Radioimmunoprecipitation assay (RIPA) Lysis Buffer (P0013B, Beyotime, Jiangsu, China). Five micrograms of total RNA were used for first strand cDNA synthesis using a cDNA Synthesis Kit (5x All-in-One RT MasterMix; Applied Biological Materials-abm, Vancouver, Canada), following the manufacturer’s instructions.

### Viral challenge and tissue collection

The WSSV virions were extracted according to previously reported methods^15,70^, and the virions were analyzed using quantitative real-time polymerase chain reaction (qPCR)^71^. The shrimp were injected with 5 × 10^7^ copies/shrimp of WSSV and phosphate-buffered saline (PBS; 140 mM NaCl, 2.7 mM KCl, 10 mM Na_2_HPO_4_, 1.8 mM KH_2_PO_4_, pH 8.0) injection was used as the control. Subsequently, shrimp hemocytes and other organs, including the heart, hepatopancreas, gills, stomach, and intestines, were collected from at least three shrimp for RNA and protein extraction at 0, 2, 6, 12, 24, 36, and 48 h post-WSSV challenge. For hemocyte collection, the hemolymph was collected from at least three shrimp using a 5 mL syringe containing 1 mL of anticoagulant (0.45 M NaCl, 10 mM KCl, 10 mM EDTA and 10 mM HEPES, pH = 7.45), and then was centrifuged at 700 × *g* for 10 min at 4 °C to collect the hemocytes for further study.

### Semi-quantitative reverse transcription PCR and quantitative real-time RT-PCR

Semi-quantitative reverse transcription PCR (RT-PCR) was used to determine the distribution of *Ampk-α/β/γ* in *M. japonicus* using specific primer pairs *Ampkα/β/γ*-RT-F and *Ampkα/β/γ*-RT-R (Supplementary Table 1). *Actb* (β-actin) was used as an internal control with primers *β-actin*-RT-F and *β-actin*-RT-R (Supplementary Table 1). The RT-PCR reaction conditions were as follows: 1 cycle at 94 °C for 3 min; 30 cycles at 94 °C for 30 s, 55 °C for 30 s, and 72 °C for 30 s; and 1 cycle at 72 °C for 10 min.

Quantitative real-time PCR (qPCR) was employed to quantify the expression patterns of *Ampkα* mRNA after WSSV challenge using a thermal cycler (qTOWER3, ANALYTIK JENA AG, Jena, Germany). The total volume of the qPCR reaction mixture was 10 μL, consisting of 5 μL of SYBR Premix Ex Taq (Takara, Shiga, Japan), 2 μl of each primer (1 μM), and 1 μL of cDNA (diluted 1:100 from original synthetized cDNA). The qPCR reaction conditions comprised: 94 °C for 5 min; 40 cycles at 94 °C for 15 s and 60 °C for 50 s; and then melting from 60 °C to 95 °C performed with a real-time thermal cycler (Bio-Rad, Hercules, CA, USA). Each experiment was repeated three times. The data were analyzed using the comparative threshold cycle (C_T_) method and the formula 2^−ΔΔCT^(ΔΔCT = CT_gene_-CT_β-actin_)^72^ using the geometric mean of the two internal control genes of *β-actin* and *Ef-1α* for normalization. The efficiency of primer pair in qPCR was analyzed following the MIQE method^73^ with a 10-fold logarithmic dilution of a cDNA mixture to generate a linear standard curve. The value obtained from control was normalized as 1, and the relative values of different time points were obtained by comparing with the control.

### Recombinant expression, purification, and antiserum preparation of *Mj*AMPKα

The sequence of *Ampkα* were amplified from intestinal cDNA using the primers AMPKα-EX-F2 and AMPKα-EX-R2 (Supplementary Table 1). *Not* I and *Sal* I restriction sites were inserted at the beginning and end of the cDNA fragments to allow the PCR products to be inserted into the *Not* I and *Sal* I restriction sites of vector pGEX-4T-2. Recombinant plasmids were transformed into *Escherichia coli* Rosetta cells, which were then cultured in Luria-Bertani medium with 0.1 mg/ml ampicillin. When the OD600 of the culture reached to 0.5, isopropyl-β-d-thiogalactopyranoside (IPTG; 0.5 mM) was added. After 4 h of culture, cells were collected by centrifugation at 8000 × *g* for 2 min. The recombinant AMPKα was expressed in an inclusion body. The AMPKα inclusion bodies were washed twice with Buffer A (50 mM Tris-HCl, 5 mM EDTA, pH 8.0) and then three times with Buffer B (50 mM Tris-HCL, 5 mM EDTA, 2 M urea, pH 8.0). The inclusion bodies were precipitated by centrifugation at 12,000 × *g* at 4 °C for 10 min. Denaturing buffer (0.1 M Tris-HCl, 10 mM DL-Dithiothreitol, 6 M guanidine hydrochloride) was added to dissolve the precipitate. The solution was incubated at 37 °C for 1 h with gentle vibration, and then centrifuged at 12,000 × *g* at 4 °C for 10 min. The collected supernatant was dialyzed in buffer (50 mM Tris-HCl, 0.5 mM EDTA, 50 Mm NaCl, 1% Glycine, 5% Glycerin) for 48 h, and then purified using GST-resin (GenScript, Nanjing, China) chromatography following manufacture’s instruction. The purified AMPK protein was used to prepare antibodies using New Zealand rabbit following the method described in our previous report^74^.

### Western blotting

The homogenates of hemocytes and other organs (heart, hepatopancreas, gills, stomach, intestines) were centrifuged at 12000 × *g* for 10 min at 4 °C. The resulting supernatant was separated by 10% SDS-PAGE and transferred to a nitrocellulose membrane with transfer buffer (25 mM Tris, 20 mM Glycine, 0.037% SDS, 20% ethyl alcohol). The membrane was blocked in 2% skim milk diluted in Tris-buffered saline (TBS) buffer for 1 h, and then incubated with primary antibodies at 4 °C overnight, including anti-AMPKα (1:500 dilution in skim milk solution), anti-virus protein 28 (VP28) (1:500 dilution), anti-β-actin (ACTB) (1:250) (prepared in our laboratory), anti-Histone-3 polyclonal antibodies (A2348, ABclonal, Wuhan, China, 1:2500); anti-phosphorylated (p)-AKT polyclonal antibodies (WLP001a, Wanleibio, Shenyang, China, 1:500), and anti-pAMPKα (YP0575, ImmunoWay, Plano, TX, USA, 1:500). The membrane was washed three times with TBST (TBS with Tween 20), and then the secondary antibody, goat anti-rabbit antibody conjugated with alkaline phosphatase (ZB2308 ZSGB-Bio, Beijing, China, 1:5000), was added and incubated for three hours at 25 °C. The membrane was finally washed three times with TBST and TBS. The immunoreactive protein bands were developed using a nitrotetrazolium blue chloride (A610379, BBI, Shanghai, China) and P-toluidine salt (A610072, BBI, Shanghai, China) solution under dark conditions or using enhanced chemiluminescence (ECL). ACTB or Histone-3 were used as controls. The densities of the protein bands and the level of phosphorylation were quantified using Image J (NIH, Bethesda, MD, USA) and expressed as a ratio to ACTB.

### RNA interference

#### Small interfering RNA

Small interfering RNA (siRNA) was prepared as described previously (http://rnaidesigner.lifetechnologies.com/rnaiexpress/)^75^, with slight modifications. DNA fragments were amplified using AMPK-α Oligo1 and AMPK-α Oligo2, AMPK-α Oligo3 and AMPK-α Oligo4, respectively (Supplementary Table 1). A T7 promoter sequence was linked to both ends of the PCR products. After extraction with phenol/chloroform and precipitation with ethanol, the DNA fragments were used as templates for siRNA synthesis. Transcription was carried out as follows: 2 μL DNA template was mixed with 2 μL 10 × transcription buffer, containing 2 μL T7 Enzyme Mix and 8 μL NTP Mix. RNase-free water was added to a total volume of 20 μL. After incubation at 37 °C for 30 min, siRNAs were incubated at 72 °C for 10 min and then cooled to room temperature for annealing. To remove the template, 17 μL of RNase-free H_2_O, 1 μL DNase I, and 2 μL RNase T1 (10 U/μl) was added and the solution was incubated at 37 °C for 5 h. After extraction with phenol/chloroform and precipitation with ethanol, the siRNAs were resuspended in 50 μL of RNase-free water. The siRNA purity and integrity were determined using agarose gel electrophoresis.

#### dsRNA

A pair of specific primers, dsCamkk-F/dsCamkk-R, dsAkt-F/dsAkt-R, and dsRictor-F/ dsRictor-R linked to the T7 promoter (Supplementary Table 1) was used to amplify a partial CaMKK/AKT/Rictor cDNA fragment using RT-PCR; *dsGfp* served as the control (Supplementary Table 1). The PCR products were extracted using the phenol/chloroform method. Afterwards, these templates were used to synthesize dsRNA. The synthesis procedure for dsRNA was as follows: A 50 μL reaction mixture comprising 2.4 μL of ATP, 2.4 μL of GTP, 2.4 μL of CTP, 2.4 μL of UTP, 4 μL of T7 RNA polymerase, 20 μL of transcription buffer, 2 μL of RNase inhibitor, 1 μL of template, and 13 μL of RNase-free water, was incubated at 37 °C for 5 h. Next, a second 50 μL mixture, containing 8 μl of DNase I, 10 μL of DNase I buffer, and 32 μL of RNase-free water, was added to the first reaction mixture and incubated at 37 °C for 1.5 h to digest the templates. The synthesized dsRNA (100 μL) was extracted with phenol-chloroform and dissolved in RNase-free water for in vivo RNAi^76^. The dsRNA was analyzed with agarose gel electrophoresis and concentration determination was performed with a UV spectrophotometer.

For the RNA interference assay, 50 μg of siRNA/dsRNA was injected into abdominal muscles of shrimp and then another 50 μg of siRNA/dsRNA was injected 36 h after the first injection. The same volume of *siGfp/dsGfp* injection was used as the control. The efficiency of RNA interference was assessed at 36 h using qPCR. After 36 h of interference, the shrimp were challenged by WSSV, and the RNA and genomic DNA was extracted from hemocytes and intestine 24 h post WSSV infection. *Vp28, Ie1* (immediate early 1) expression and WSSV copies was detected by qPCR.

### Injection of the AMPK activator, AICAR

AICAR, a specific activator of AMPK^77^, was used to activate AMPK and further analyze the function of AMPK during WSSV infection. Different concentrations of AICAR (75 μg, 150 μg, and 250 μg/g shrimp) were firstly injected into shrimp for analyzing their effects on shrimp viability. WSSV replication (using *Vp28* expression as the indicator) was also detected in the shrimp following WSSV infection. We chose 150 μg/g shrimp injection for further experiments. AICAR-injected shrimp were challenged by WSSV (5 × 10^7^ virions) at 2 h post AICAR injection, and then the hemocytes and intestines were collected from the shrimp to detect the phosphorylation of AMPKα, the expression of *Vp28* and *Ie1*, and the viral copy number. At the same time, proteins were extracted from hemocytes and intestines to detect the level of VP28.

### Injection of an AMPK inhibitor, Dorsomorphin 2HCL

Dorsomorphin/Compound C (C_24_H_25_N_5_O; 6-[4-(2-piperidin-1-ylethoxy) phenyl]-3-pyridin-4-ylpyrazolo [1, 5-a] pyrimidine) is an AMPK inhibitor^78^. Dorsomorphin 2HCL was used to inhibit AMPK activity to further analyze AMPK function in WSSV infection. Different concentrations of Dorsomorphin 2HCL (2.5 μg, 5 μg, and 10 μg/g shrimp) were firstly injected it into shrimp to observe their effects on shrimp viability. WSSV replication (using *Vp28* expression as the indicator) was also detected in the shrimp following WSSV infection at 2 h post activator injection. We chose 5 μg/g shrimp injection for further experiments. The shrimp were challenged by WSSV (5 × 10^7^ virions) at 2 h post Dorsomorphin 2HCL injection, and then hemocytes and intestines were collected from the shrimp to detect the phosphorylation of AMPKα, *Vp28* and *Ie1* gene expression, and the viral copy number. At the same time, proteins were extracted from hemocytes and intestines to detect the level of VP28.

### Injection of an mTORC1 inhibitor, Rapamycin, and an mTORC1/2 inhibitor, Torin1

Rapamycin binds to TOR in an allosteric manner with the FK506-binding protein 12 (FKBP12) to inhibit mTORC1, but not mTORC2 activities^79^. The mTOR ATP-competitive inhibitor Torin1 has been reported acting on mTOR both complexes, mTORC1 and mTORC2^80^. Rapamycin (Abmole, Houston, 40 ng/g shrimp) or Torin1 (20 μg/g shrimp, Abmole, Houston) was injected into shrimp, then the shrimp were challenged by WSSV (5 × 10^7^ virions) at 2 h post Rapamycin or Torin1 injection. The hemocytes and intestines were collected from the shrimp at 24 h post WSSV infection, AKT phosphorylation and WSSV replication in the shrimp were analyzed. Same dose injection of DMSO was used as control.

### Survival rate assay

The AMPK activator, AICAR, AMPK inhibitor, Dorsomorphin 2HCL, and calcium chelator, BAPTA, *Camkk*-RNAi, and *Akt*-RNAi were used in the shrimp survival rate assay. First, shrimp in triggering the Warburg-like effect were randomly divided into two groups (30 shrimp/group) comprising the experimental group injected with AICAR/Dorsomorphin 2HCL/BAPTA/*dsCamkk*/*dsAkt* and the control group injected with same volume of dimethyl sulfoxide (DMSO) or *dsGfp*. At 2 h after injection of AICAR/Dorsomorphin 2HCL/BAPTA/DMSO or at 24 h after injection of *dsCamkk* /*dsAk* /*dsGfp*, shrimp were injected with WSSV (1 × 10^7^) for virus infection. The mortality of the shrimp in both groups was observed every 12 h after WSSV infection. The survival rate of each group was calculated and the survival curves are presented as Kaplan-Meier plots. Differences between the two groups were analyzed using the log-rank test in the software GraphPad Prism 8.0 (GraphPad Inc., La Jolla, CA, USA).

### Calcium ion detection

To determine whether WSSV infection affects the concentration of calcium ions, we used the intestine tissue of shrimp to detect the free calcium ions using a Calcium Colorimetric Assay Kit (Beyotime Biotechnology, Shanghai, China). Shrimp intestines were collected at 0, 10, 20, and 30 min, and 1, 2, 6, and 12 h post WSSV challenge for calcium detection. The intestine tissue (30 mg) was homogenized in 300 μL of sample lysis buffer using a glass homogenizer, followed by centrifugation at 14,000 × *g* for 5 min. First, the protein concentration of the supernatant was measured using the Bradford method. The supernatant was placed on ice for the subsequent measurement. Next, the calcium standard solution was diluted to different concentrations to create a standard curve. The concentration of the supernatant was detected following manufacture’ protocols using a microplate reader (TECAN M200 PRO, Tecan Group Ltd., Männedorf, Switzerland) at 575 nm.

### Injection of BAPTA, a Ca^2+^ chelator

To further verify that WSSV can activate AMPK through calcium signaling pathways, we injected the Ca^2+^ chelator BAPTA (1,2-bis(o-aminophenoxy) ethane-N, N, N′,N′-tetraacetic acid) and detected the phosphorylation levels of AMPK and AKT using a human anti-p-AMPKα1/α2(Thr^183/172^) antibody (YP0575, ImmunoWay) and a human anti-p-AKT (Ser^473^) antibody (WLP001a, Wanleibio). After infection with WSSV, BAPTA (7 μg/g shrimp, Abmole, Houston) was injected into the shrimp and the same volume of DMSO injection was used as the control. Shrimp intestines were collected at 1 h post BAPTA injection for calcium detection using a Calcium Crimson™, AM kit (Invitrogen, Waltham, MA, USA) following manufacture’s instruction. The intestines were incubated with 3 μM Calcium dye at 37 °C for 30 min, and then washed with PBS three times for 5 min each time. The treated intestines were placed on a slide with glycerin, covered with a cover slip, and then observed under a fluorescence microscope (Olympus BX51, Tokyo, Japan). The optical density of the fluorescence image was measured using ImageJ.

### Immunocytochemical assay

To detect the subcellular distribution of AMPK in shrimp hemocytes challenged by WSSV, an immunocytochemical assay was performed following a previously reported method^81^. Shrimp Hemocytes were collected in 4% paraformaldehyde and anticoagulation mixtures (1:1) at 0, 20 min, 30 min, and 1 and 2 h after WSSV challenge. The hemocytes were left on ice for 10 min and the then washed three times with PBS, centrifuged at 700 × *g* for 5 min at 4 °C. After re-suspending in PBS, the hemocytes were dropped onto poly-lysine coated glass slides and left to stand for 30 min. The slides were washed six times with PBS, and left to stand for 10 min. After treatment with 0.2% Triton X-100, the hemocytes were washed with PBS four times, and blocked with 3% bovine serum albumin (dissolved in PBS) for 30 min at 37 °C. Anti-AMPKα antibody was then added (1:100 diluted in 3% bovine serum albumin) and the cells were incubated over night at 4 °C. The hemocytes were washed with PBS five times, incubated with goat anti-rabbit antibody conjugated with ALEXA 488 (1:1000 diluted in PBS) (A23220, Abbkine, Wuhan, China) for 1 h at 37 °C, washed with PBS five times, and then stained with 4-6-diamidino2-phenylindole (DAPI) for 10 min at room temperature. After washing five times, the slides were examined under a fluorescent microscope (Olympus BX51).

### Isolation of nuclear and cytoplasmic proteins

The intestine tissue was dissected from shrimp and washed in PBS three times. Protein extraction was carried out by using a nuclear and cytoplasmic protein extraction kit (R0050, Solarbio, Beijing, China) following the manufacturer’s instructions. At least three shrimp were used for protein extraction to eliminate individual differences.

### Hexokinase and Pyruvate kinase activity measurements

Hexokinase (HK) is the first key enzyme in the intersection point of glycolysis and pentose phosphate pathways^82^. Pyruvate kinase (PK) is one of the main rate-limiting enzymes in glycolysis^83^. Enzyme activity was detected using a Hexokinase Assay Kit or a Pyruvate kinase Assay Kit (Solarbio Science and Technology, Beijing, China), following the manufacturer’s instruction. Briefly, hemocytes were collected using above mentioned method and counted by using a hemacytometer under a microscope, number of blood cells (10^4^): volume of extraction buffer (mL) was 500:1, and the intestines were weighed using a microbalance, the tissue mass (g): volume of extraction buffer (mL) is 1:10. Hemocytes or intestines from *Ampka*-knockdown shrimp were collected and mixed with extraction buffer at a ratio of 500:1 or 1:10, and homogenized on ice. After centrifugation at 8000 × *g* at 4 °C for 10 min, the supernatant was used for activity detection. The HK and PK activities in hemocytes and intestines of *Ampka*-knockdown shrimp were measured using a Hexokinase Assay Kit and a Pyruvate kinase Assay Kit (Solarbio Science and Technology, Beijing, China), according to the manufacturer’s instruction. The absorbance of HK and PK was measured at 340 nm. The enzymic activity of hemocytes was defined as a unit of enzymic activity based on the production of 1nmol NADPH per 10^4^ cells per minute, while the enzymic activity of small intestine was defined as a unit of enzymic activity based on the production of 1nmol NADPH per g tissue per minute. The value of activity of HK or PK in control group (*siGfp* injection, Dorsomorphin 2HCL, PBS) was normalized to 1.0 for comparisons.

### Pyruvate, ATP, and lactic acid detection assay

The Pyruvate (PA), ATP, and lactic acid (LA) assay kits (Solarbio Science and Technology) were used to detect the PA, ATP, and LA contents in hemocytes (cell number was counted using a hemacytometer) and intestine tissues of *Ampkα*-knockdown shrimp. The intracellular pyruvate content was detected via absorption at 520 nm using the pyruvate assay kit (Solarbio Science and Technology) according to the manufacturer’s instructions. The intracellular ATP content was detected via absorption at 340 nm using the ATP assay kit (Solarbio Science and Technology) following the manufacturer’s instructions. The lactic acid production in the culture supernatant was measured at 570 nm using a lactic acid assay kit (Solarbio Science and Technology) following manufacturer’s instructions.

### Electrophoretic mobility shift assay

EMSA was performed to investigate binding of rHIF1α to the binding site in promoter region of hexokinase (HK). The recombinant DNA binding domain of HIF1α protein was expressed in *E. coli* with pGEX-4T-2/Hif1α. The *hexokinase* promoter sequence was obtained from genome of *M. japonicus* online. Oligonucleotides of wild HK probes from the promoter (sense 5′- CAAATGACTAGAACGTGCGACATTAGTGAA-3′ and antisense 5′- TTCACTAATGTCGCACGTTCTAGTCATTTG -3′) and mutant probes (sense 5′- CAAATGACTAGACACAGCGACATTAGTGAA -3′ and antisense 5′- TTCACTAATGTCGCTGTGTCTAGTCATTTG -3′) were synthesized, and the sense probes were biotinylated by Sangon Biotech Company (Shanghai, China). The HK probes were diluted to 10 μM with annealing buffer (Solarbio, China). Equimolar sense and antisense probes were annealed at 95 °C for 10 min and cooled down slowly to room temperature to create double-stranded probes. EMSA was performed according to the instruction of Chemiluminescent EMSA Kit (Beyotime, China). Briefly, different amounts of purified rHIF1α (1 and 3 μg) were used in the EMSA analysis, the rGST (1 and 3 μg GST) was used as control. Mutant and wild type HK probes (with final concentrations of 0.1 and 1 μM) were firstly incubated with 3 μg HIF1α at 25 °C for 10 min, respectively. Then all samples were mixed with biotinylated HK probes (with final concentrations of 0.1 μM) and incubated at 25 °C for 20 min. After separation in a native 6% polyacrylamide gel, the samples were transferred to a Hybond N + nylon membrane, followed by crosslinking under UV light. Then, the membrane was blocked and incubated in HRP-labeled streptavidin following the instruction of Chemiluminescent EMSA Kit. The membrane band was developed by ECL system.

### Statistics and reproducibility

The data are presented as the mean ± standard deviation (SD) of at least three independent experiments. Significance differences were analyzed using Student’s *t*-test for paired comparisons or one-way ANOVA for multiple comparisons. Asterisks in figures indicate statistical significance (*P < 0.05; **P < 0.01; ***P < 0.001). The different lowercase letters indicate significant differences (*P* < 0.05) in the ANOVA analysis. The nonparametric test, Mann-Whitney *U* test was also used for the un-normally distributed data. For the survival rate analysis, the survival curves were presented as Kaplan-Meier plots. Differences for multiple comparisons were analyzed using the log-rank test in GraphPad Prism 8.0 and statistical significance was accepted at *P* < 0.05. Western blotting bands were analyzed based on three independent replicates using ImageJ software (National Institutes of Health, http//imagej.nih.gov/ij/download.html).

### Reporting summary

Further information on research design is available in the Nature Portfolio Reporting Summary linked to this article.

## Supplementary information

Supplementary Information

Description of Additional Supplementary Files

Supplementary Data 1

Reporting Summary

## Data Availability

All of the data generated or analyzed during this study are included in this published article and its supplementary information files. The numerical source data for graphs and charts are available in Supplementary Data 1.
