# Supplementary material for: WSSV exploits AMPK to activate mTORC2 signaling for proliferation by enhancing aerobic glycolysis
Source: Commun Biol. 2023 Apr 3;6:361. doi: 10.1038/s42003-023-04735-z (PMC10070494; doi:10.1038/s42003-023-04735-z)
Supplement: Supplementary file 1 — Supplementary Information [file 42003_2023_4735_MOESM1_ESM.pdf]

Supplementary Materials

WSSV exploits AMPK to activate mTORC2 signaling for viral proliferation by enhancing aerobic glycolysis

Peng Zhang[1#], Hai-Jing Fu[1#], Li-Xia Lv[1], Chen-Fei Liu[1], Chang Han[1], Xiao-Fan Zhao[1], Jin-Xing Wang[1,2,*]

The PDF contains:

Supplementary Figure 1-9

Supplementary Table 1

Uncropped and unedited blot images: Supplementary Figure 10-33

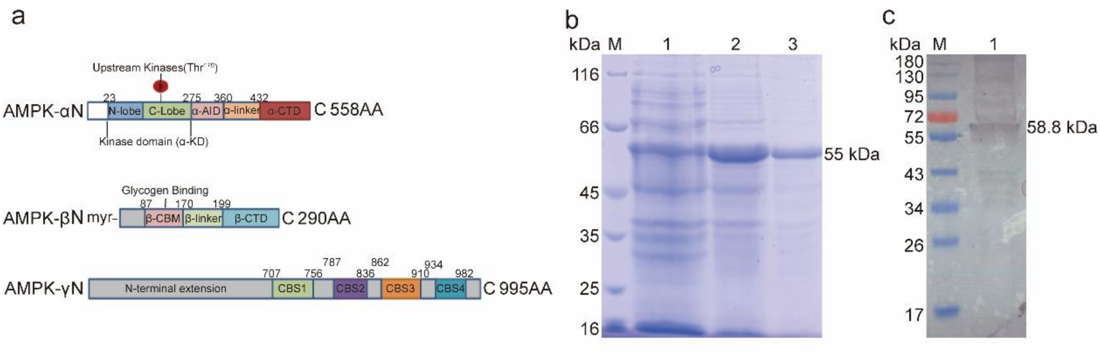

Supplementary Figure 1 Domain architectures of the AMPK complex and antibody preparation of AMPKα. a The domain architectures of three AMPK subunits from *M. japonicus*. **b** Recombinant expression in *E. coli* and purification of AMPKα. Lane 1, total proteins of *E. coli* with AMPKα-pGEX4T-2 without IPTG induction; Lane 2, Total proteins of the *E. coli* with IPTG induction; Lane 3, Purified recombinant AMPKα; lane M, protein molecular mass markers. **c** Western blotting using AMPKα polyclonal antibodies to detect AMPKα in normal hemocytes of shrimp. Lane M, protein molecular mass markers; Lane 1, the native AMPKα in hemocytes detected using AMPKα polyclonal antibodies.

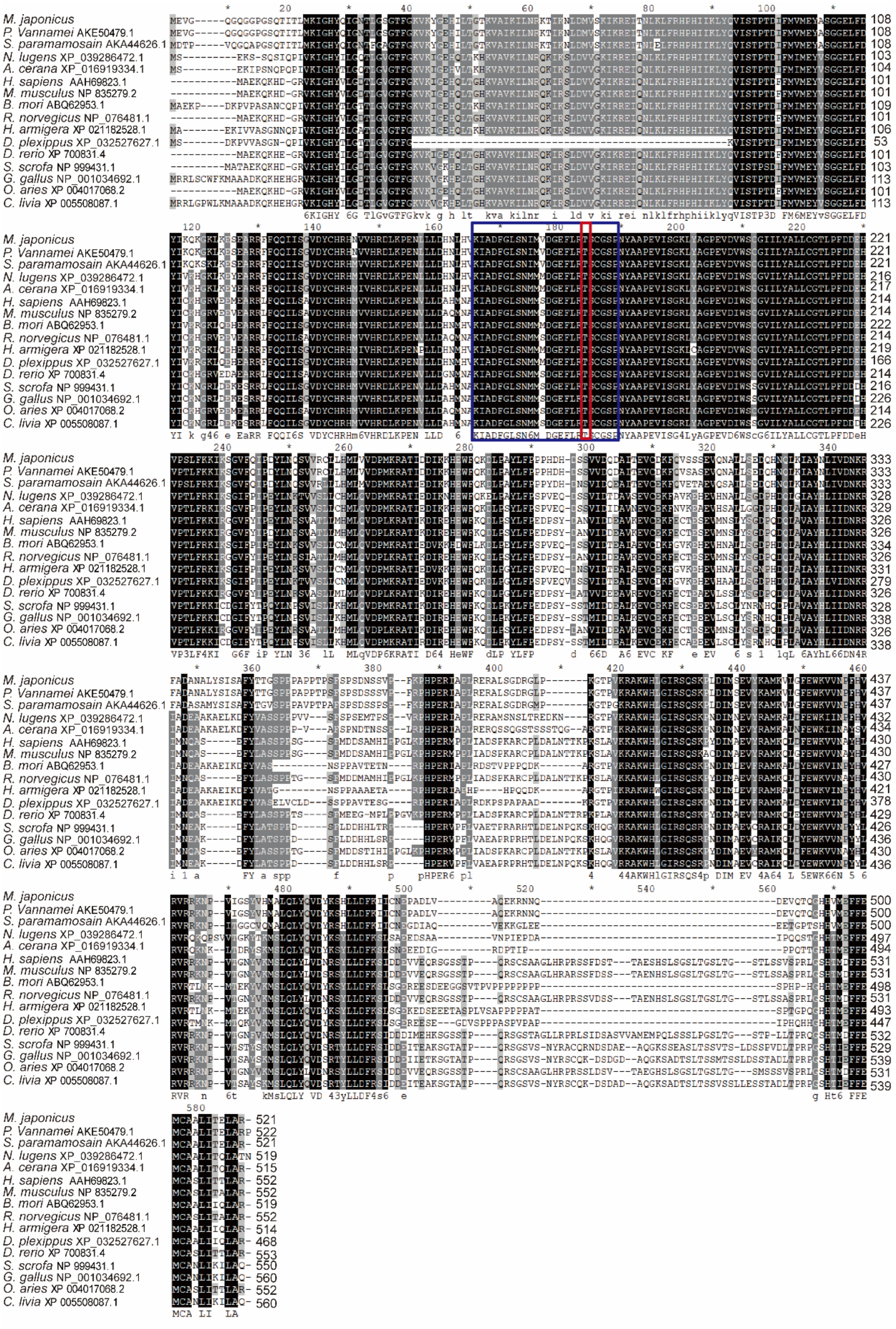

Supplementary Figure 2 The amino acid sequence alignment of AMPKα proteins from different species. Blue Boxed residues show the conserved activation ring. Red Boxed residues show the conserved phosphorylation sites.

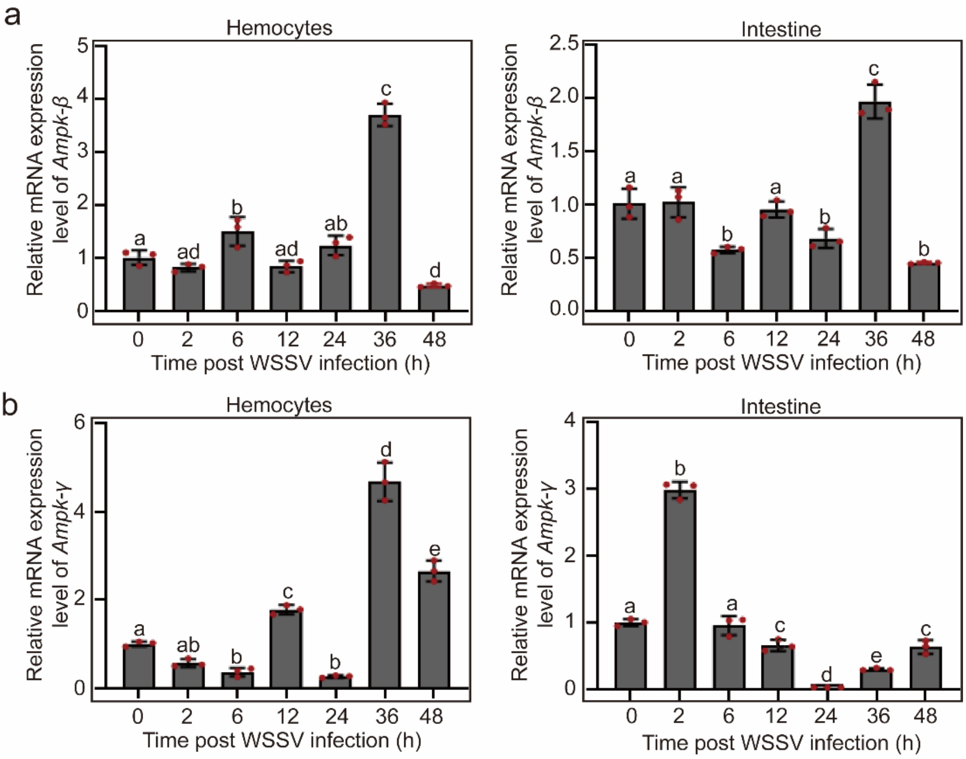

Supplementary Figure 3 Expression profiles of AMPKs in shrimp challenged by WSSV. a The expression patterns of *Ampkβ* in hemocytes and intestine of the shrimp analyzed using qPCR. **b** The expression patterns of *Ampkγ* in hemocytes and intestine of the shrimp. All results shown are means ± SD for experiments performed at least three times. Multiple different comparisons were analyzed with log-rank test using the GraphPad Prism 8.0 software and a significant difference was accepted at $P < 0.05$.

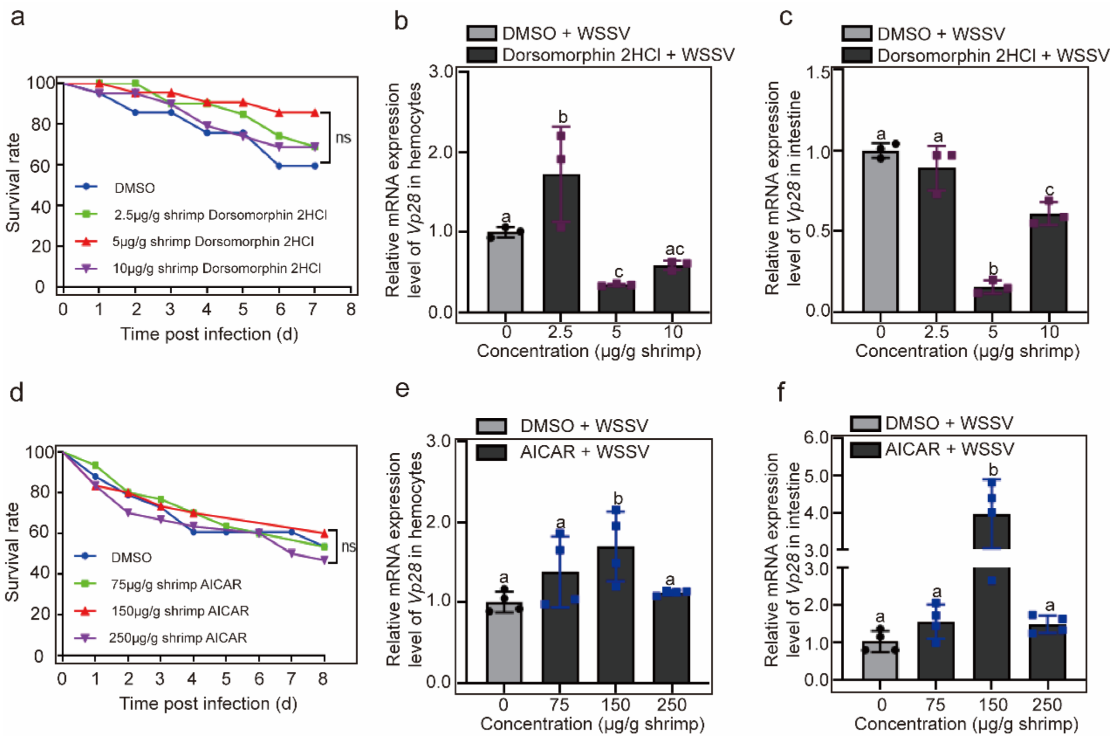

Supplementary Figure 4 Survival analysis of shrimp injected with different concentrations of Dorsomorphin 2HCL and AICAR, and WSSV replication analysis in the shrimp following WSSV infection. a Survival rate of shrimp injected with different concentrations of Dorsomorphin 2HCL. **b, c** *Vp28* expression in hemocytes (**b**) and intestine (**c**) of shrimp injected with different concentrations of Dorsomorphin 2HCL following WSSV infection. Shrimp injected with the same amount of DMSO were used as controls. **d** Survival rate of shrimp injected with different concentrations of AICAR. **e, f** *Vp28* expression in hemocytes (**e**) and intestine (**f**) of shrimp injected with different concentrations of AICAR following WSSV infection. Shrimp injected with the same amount of DMSO in each group were used as controls. All results shown are means ± SD for experiments performed at least three times. Multiple different comparisons were analyzed with log-rank test using the GraphPad Prism 8.0 software and a significant difference was accepted at $P < 0.05$.

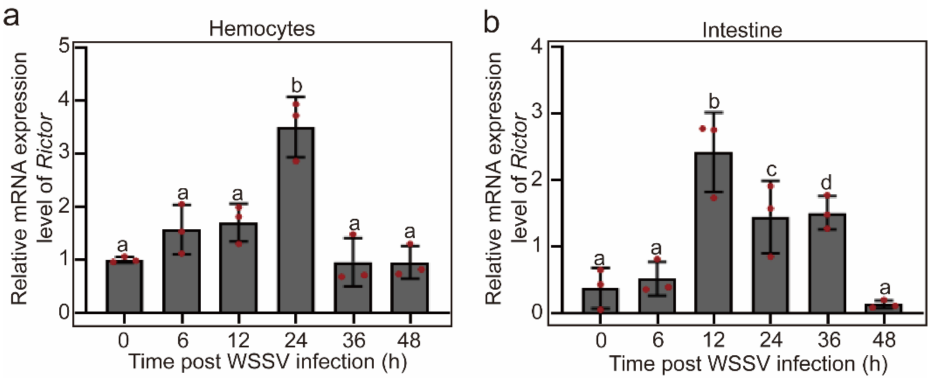

Supplementary Figure 5 Expression profiles of Rictor in hemocytes (a) and intestines (b) of shrimp challenged by WSSV analyzed using qPCR. All results shown are means ± SD for experiments performed at least three times. Multiple different comparisons were analyzed with log-rank test using the GraphPad Prism 8.0 software and a significant difference was accepted at $P < 0.05$.

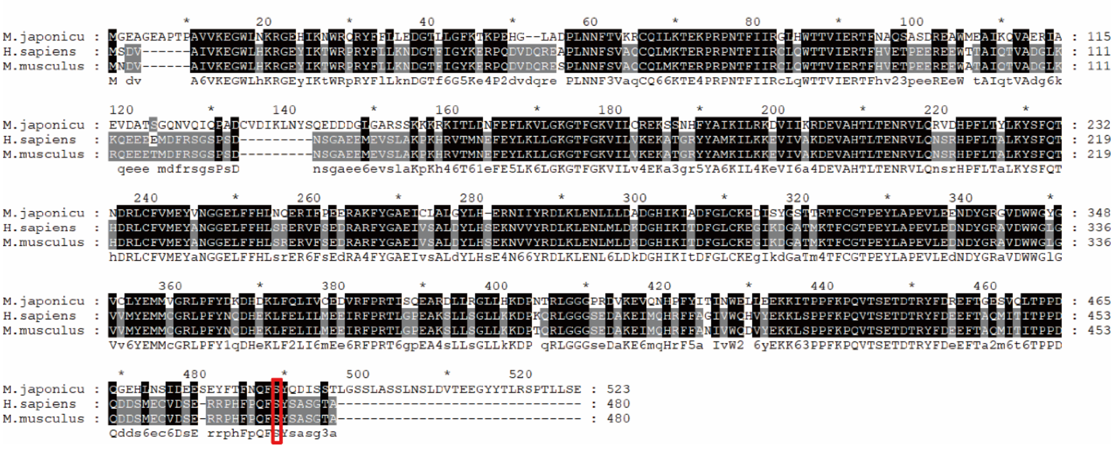

Supplementary Figure 6 Alignment of shrimp AKT amino acid sequence with AKTs of other species. The conserved phosphorylation sites were boxed in red.

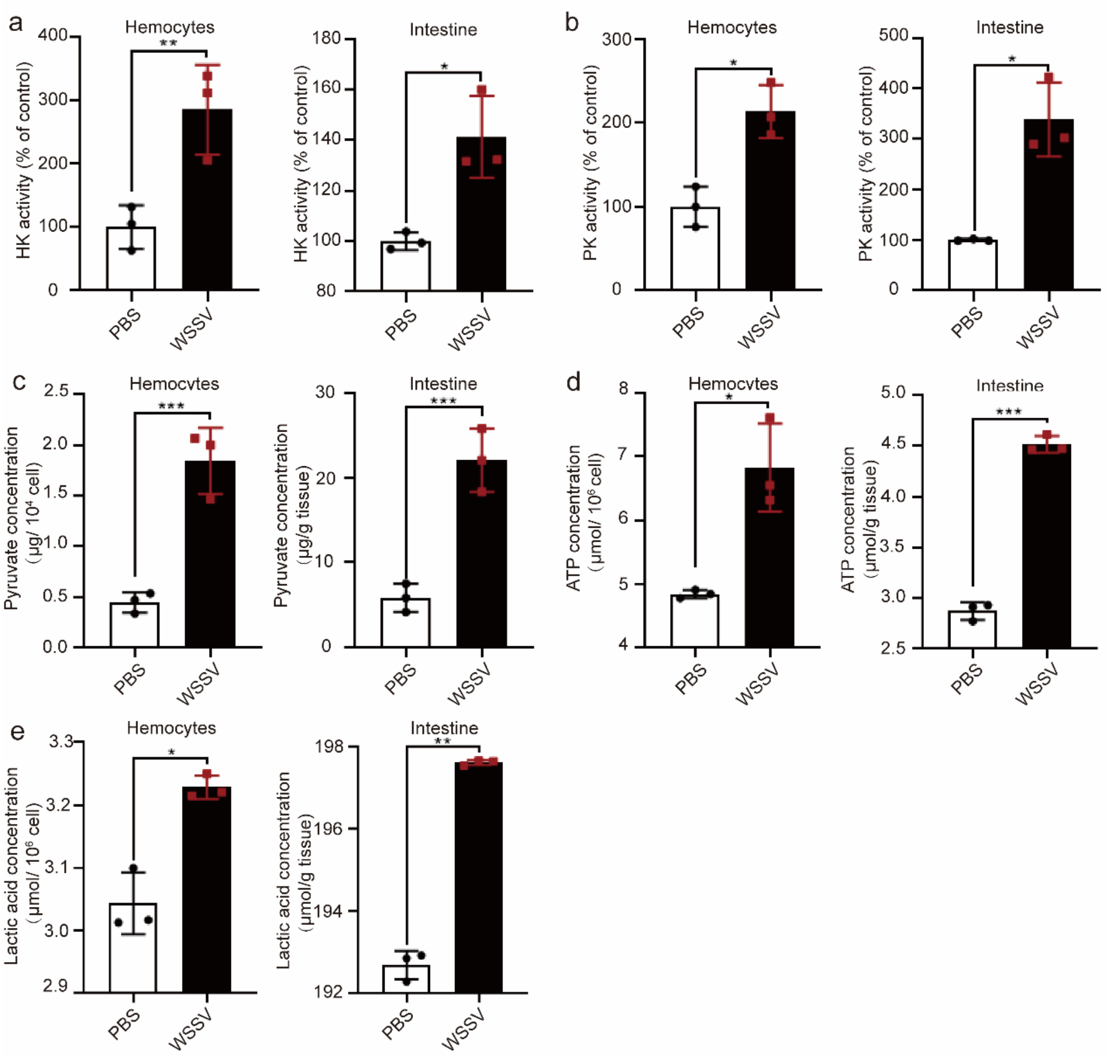

Supplementary Figure 7 Changes in the enzyme activities and glycolytic metabolites during WSSV infection in shrimp. a HK activity analysis in hemocytes and intestine of shrimp challenged by WSSV, PBS injection was used as a control. **b** PK activity analysis in hemocytes and intestine of shrimp challenged by WSSV, PBS injection was used as a control. **c** Pyruvate level analysis in hemocytes and intestine of shrimp challenged by WSSV, PBS injection was used as a control. **d** ATP level analysis in hemocytes and intestine of shrimp challenged by WSSV, PBS injection was used as a control. **e** LA level analysis in hemocytes and intestine of shrimp challenged by WSSV, PBS injection was used as a control. All results are shown by means ± SD for experiments were performed at least three times; the data were analyzed statistically using Student's t test. *, $P < 0.05$; **, $P < 0.01$; ***, $P < 0.001$.

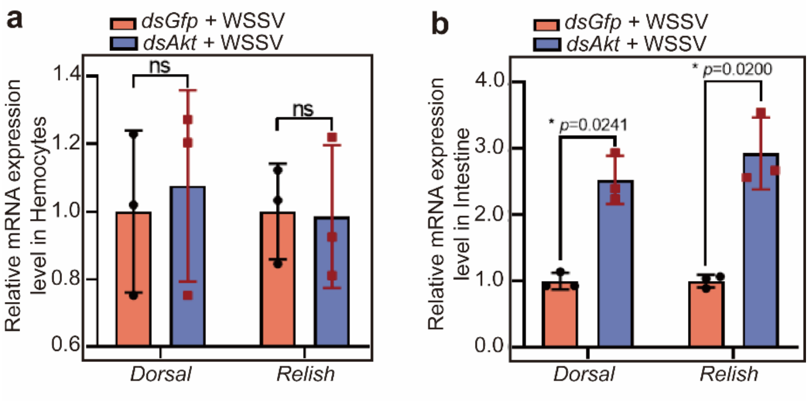

Supplementary Figure 8 The expression of *Dorsal* and *Relish* in *Akt*-knockdown shrimp challenged by WSSV analyzed by qPCR. a. Hemocytes, b. Intestine. All results are shown by means ± SD for experiments were performed at least three times; the data were analyzed statistically using Student's t test. *, $P < 0.05$; **, $P < 0.01$; ***, $P < 0.001$.

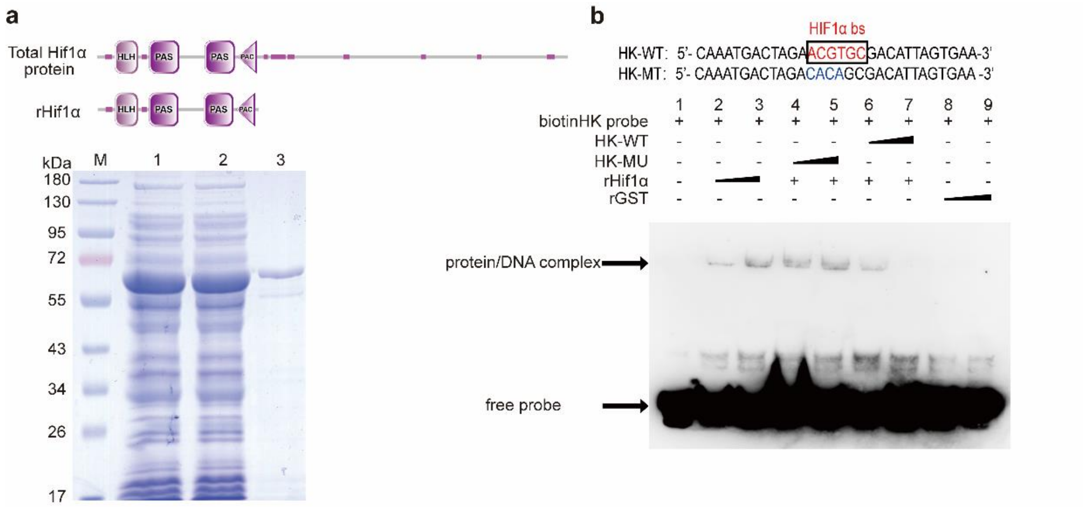

Supplementary Figue 9 The binding activity of rHIF1α to binding site in HK promoter region was detected by EMSA *in vitro*. **a** Domain architectures of HIF1α and recombinant expression in *E. coli* and purification of HIF1α. Lane 1, total proteins of *E. coli* with pGEX4T-2/HIFα with 0.1 mM IPTG induction; Lane 2, Total proteins of the *E. coli* with 0.5 mM IPTG induction; Lane 3, Purified recombinant HIFα; lane M, protein molecular mass markers. **b** Upper panel: synthesized probe sequences containing binding site from promoter sequence of HK. Lower panel: EMSA was performed to validate the interaction of rHIF1α with its binding site in HK promoter. Lane 1: negative group (without rHIF1α); Lane 2-3: positive groups (1, 3 μg rHIF1α); Lane 4-5: mutant HK group (3 μg rHIF1α incubated with two different concentrations of mutant HK probes; HK-MU, mutant HK probes without biotin); Lane 6-7: wild HK group (3 μg rHIF1α incubated with two different concentrations of wild HK probes; HK-WT, wide HK probes without biotin); Lane 8-9: GST control protein (1, 3 μg rGST).

Supplementary Table 1. Sequences of primers used in the present study

Primers	Sequence (5’-3’)	Gene target and GenBank number	Tm[1] (℃)	A[2] (bp)	Efficiency[3] (%)
**RT-PCR and qPCR**					
IE1-RT-F	GACTCTACAAATCTCTTTGCCA	*Ie1*,	51.9	502	123.57
IE1-RT-R	CTACCTTTGCACCAATTGCTAG	AF332093	50.8		
VP28-RT-F	AGCTCCAACACCTCCTCCTTCA	*Vp28*,	60.6	162	101.20
VP28-RT-F	TTACTCGGTCTCAGTGCCAGA	AY422228	57.8		
β-actin-RT-F	AGTAGCCGCCCTGGTTGTAGAC	*Actb*,	55.3	240	107.78
β-actin-RT-R	TTCTCCATGTCGTCCCAGT	GU645235	54.6		
Ef-1α-RT-F	GGATTGCCACACCGCTCACA	*Ef1a*,	61.4	223	98.83
Ef-1α-RT-R	CACAGCCACCGTTTGCTTCAT	AB458256	58.9		
AMPKα-RT-F	ACAAGCGTTTTGCTGATGC	*Ampka*,	57.0	156	101.56
AMPKα-RT-R	CTCGCAAAGGTGCTATTCTC	OL364937	55.3		
AMPKβ-RT-F	GATGGCAAAGATAGTGAAGAGC	*Ampkb*,	56.2	196	116.83
AMPKβ-RT-R	GTAGGTCCACAATGGCAACA	OL364938	56.3		
AMPKγ-RT-F	TCAAACGCCTCACGGAAAC	*Ampkr*	59.1	284	126.91
AMPKγ-RT-R	CGATGTAGCGGCAAGAATGT	XP042865543	58.7		
CAMKK-RT-F	TGATGGGGACGTGGAAGAA	*Camkk*	58.9	216	108.27
CAMKK-RT-R	CGACACCGGATACTTCAGGA	XP042859770	58.1		
HK-RT-F	GCTTAGATTTCATCCGCACAG	*Hk*,	57.8	283	89.40
HK-RT-R	CAGTCAGCGTCAGTAGCATTG	OL364939	56.8		
PFK-RT-F	GGAGAAGTTGCCGTATTTGA	*Pfk*,	55.4	178	83.73
PFK-RT-R	CACGATAGTCTGGGCTGTTT	OL364940	55.1		
PK-RT-F	CTCGTGGTGATTTGGGAATT	*Pk*,	56.3	255	92.88
PK-RT-R	TGAGTCAGTGGGCAGATGGA	OL364941	56.9		
Glut1-RT-F	AACTGCGGACTGAACACCTC	*Glut1*,	54.7	162	93.60
Glut1-RT-R	TCTTCGTAGGGCTCTTCGTG	OL364942	56.9		
HIF1α-RT-F	CCCAGAAGAACAGCGAGAA	*Hif1a*	54.7	171	102.30
HIF1α-RT-R	TCAGGTAGGCAATGGTAAGC	XP042868035	54.5		
Rictor-RT-F	TCAGGGCAGTGTTATGTTGG	*Rictor*,	55	162	81.72
Rictor-RT-R	AAGGCGAGGTTAAGGAAAAG	OK143319	52.2		
AKT-RT-F	GTTGACTGGTGGGGTTATGGA	*Akt*,	57.1	243	106.06
AKT-RT-R	GGTGATGTAGAAGGGGTGATT	KP419299	54.0		
**Recombinant expression**					
AMPKα-EX-F	TACTCAGTCGACATGGAGGTCGGCCAGGGA				
AMPKα-EX-R	TACTCAGCGGCCGCTTAACGAGCTAGTTCTGT				
AMPKα-EX-F2	TACTCAGTCGACGGTCATTACCAGATCGGA				
AMPKα-EX-R2	TACTCAGCGGCCGCCTTCTGGAACCATTCATG				
**SiRNA**					

**Primers**	**Sequence (5’-3’)**
GFP- Oligo1	GATCACTAATACGACTCACTATAGGGGGAGTTGTCCCAATTCTTGTT
GFP- Oligo2	AACAAGAATTGGGACAACTCCCCCTATAGTGAGTCGTATTAGTGATC
GFP- Oligo3	AAGGAGTTGTCCCAATTCTTGCCCTATAGTGAGTCGTATTAGTGATC
GFP- Oligo4	GATCACTAATACGACTCACTATAGGGCAAGAATTGGGACAACTCCTT
AMPKα-Oligo1	GATCACTAATACGACTCACTATAGGGGCTGCACCAGAAGTTATATTT
AMPKα-Oligo2	AAATATAACTTCTGGTGCAGCCCCTATAGTGAGTCGTATTAGTGATC
AMPKα-Oligo3	AAGCTGCACCAGAAGTTATATCCCTATAGTGAGTCGTATTAGTGATC
AMPKα-Oligo4	GATCACTAATACGACTCACTATAGGGATGGCCGTCTGGTTCTTGCTT
AMPKβ-Oligo1	GATCACTAATACGACTCACTATAGGGCCTACTCTCCAGGGAGAATTT
AMPKβ-Oligo2	AAATTCTCCCTGGAGAGTAGGCCCTATAGTGAGTCGTATTAGTGATC
AMPKβ-Oligo3	AACCTACTCTCCAGGGAGAATCCCTATAGTGAGTCGTATTAGTGATC
AMPKβ-Oligo4	GATCACTAATACGACTCACTATAGGGATTCTCCCTGGAGAGTAGGTT
**RNAi**	

GFP-Ri-F	TAATACGACTCACTATAGGGGGGTGTACAGCTCGTCCATGC
GFP-Ri-R	TAATACGACTCACTATAGGGCTTGTACAGCTCGTCCATGC
CAMKK-Ri-F	GCGTAATACGACTCACTATAGGGCAATGGGCGTGACCTTAT
CAMKK-Ri-R	GCGTAATACGACTCACTATAGGTCAAGCGACCTCTTCCTGT
Rictor-Ri-F	GCGTAATACGACTCACTATAGGGAAGCAGATGGGAAAAATA
Rictor-Ri-R	GCGTAATACGACTCACTATAGGCAAGCGGAATGAATAGGAA
Raptor-Ri-F	GCGTAATACGACTCACTATAGGGAGACATAATGGAACAGAAG
Raptor-Ri-R	GCGTAATACGACTCACTATAGGTTGGATGAAAGAGTAACACA
AKT-Ri-F	GCGTAATACGACTCACTATAGGGGGCGAGAAGCTCAAAGAA
AKT-Ri-R	GCGTAATACGACTCACTATAGGAACCCCACCAGTCAACACCT

1. Annealing temperature (°C)

2. Amplicon size (bp)

3. qPCR assay efficiencies of related genes (%)

:

Uncropped and unedited blot/gel images

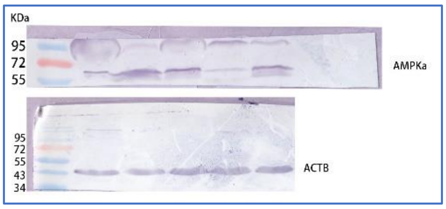

Supplementary Figure 10. Western blots of Figure 1b. The blot image is from two blot membranes.

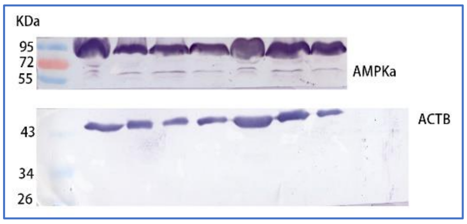

Supplementary Figure 11. Western blots of Figure 1e. The blot image is from two blot membranes.

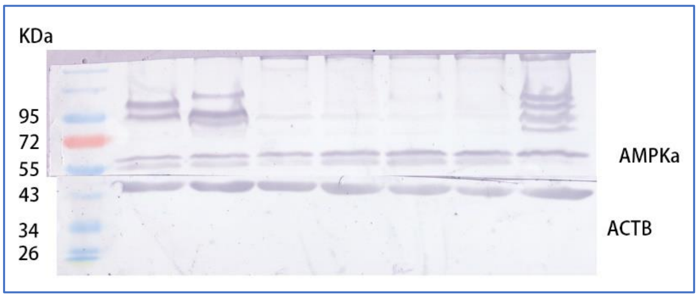

Supplementary Figure 12. Western blots of Figure 1f. The blot image is from the same blot membrane.

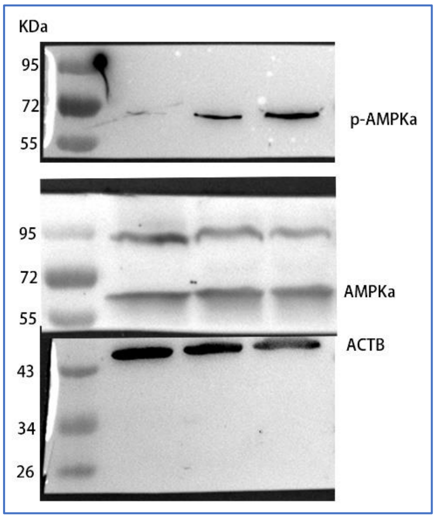

Supplementary Figure 13. Western blots of Figure 1g. The blot image is from three blot membranes.

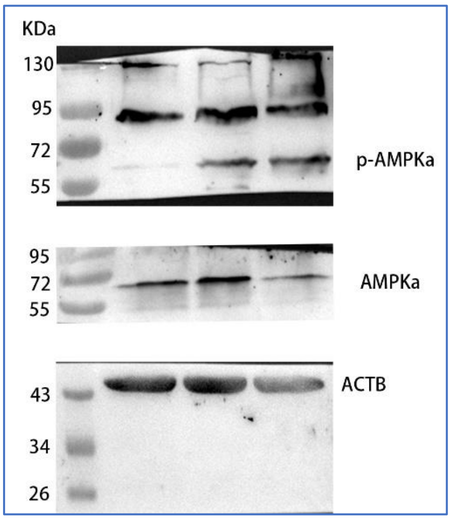

Supplementary Figure 14. Western blots of Figure 1h. The blot image is from three blot membranes.

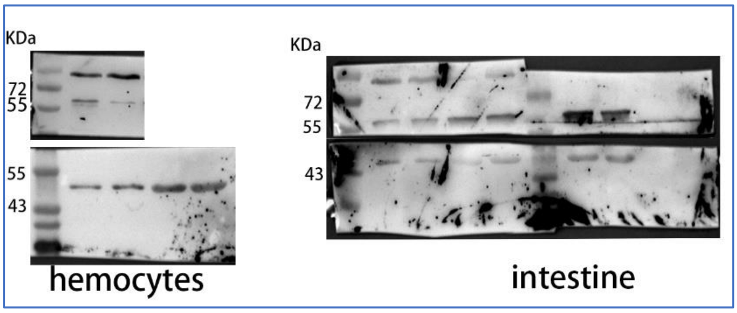

Supplementary Figure 15. Western blots of Figure 2b. The blot image is from three blot membranes.

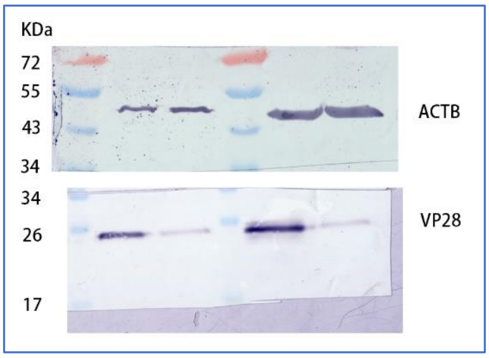

Supplementary Figure 16. Western blots of Figure 2d. The blot image is from two blot membranes.

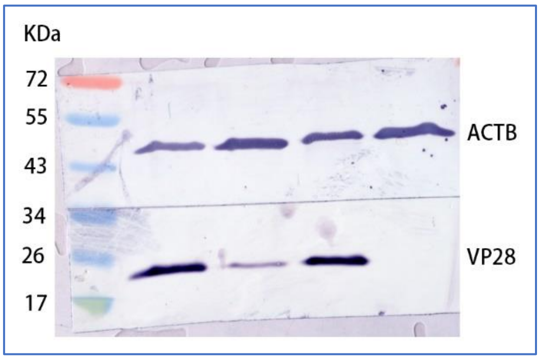

Supplementary Figure 17. Western blots of Figure 2i. The blot image is from the same blot membrane.

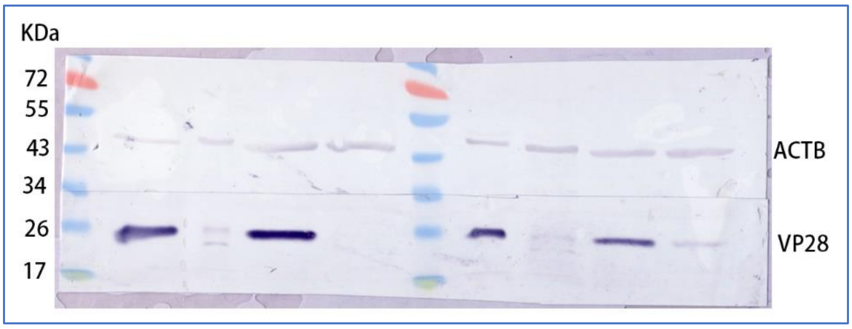

Supplementary Figure 18. Western blots of Figure 3b. The blot image is from the same blot membrane.

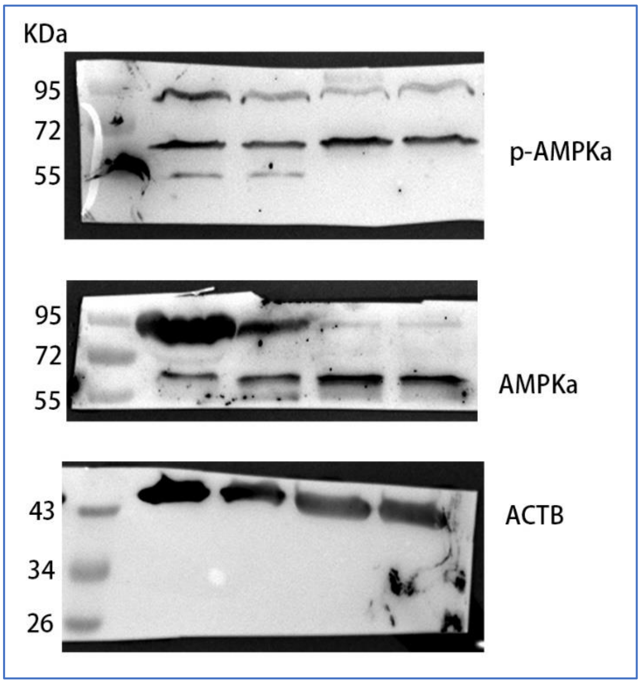

Supplementary Figure 19. Western blots of Figure 3c. The blot image is from three blot membranes.

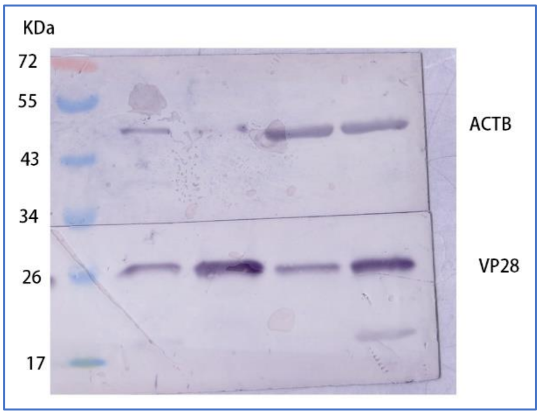

Supplementary Figure 20. Western blots of Figure 3f. The blot image is from the same blot membrane.

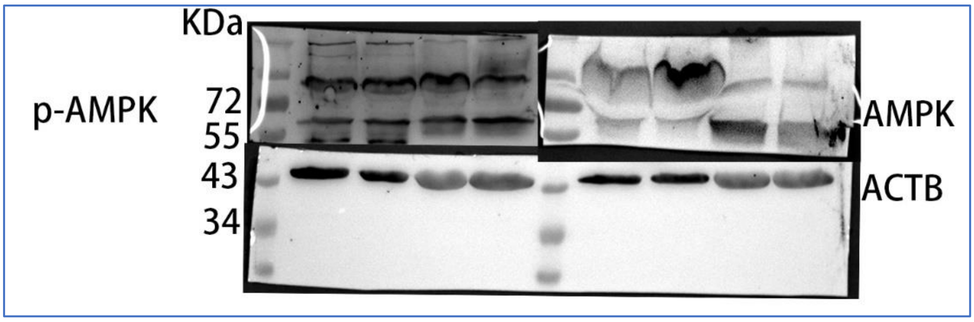

Supplementary Figure 21. Western blots of Figure 3g. The blot image is from the same blot membrane.

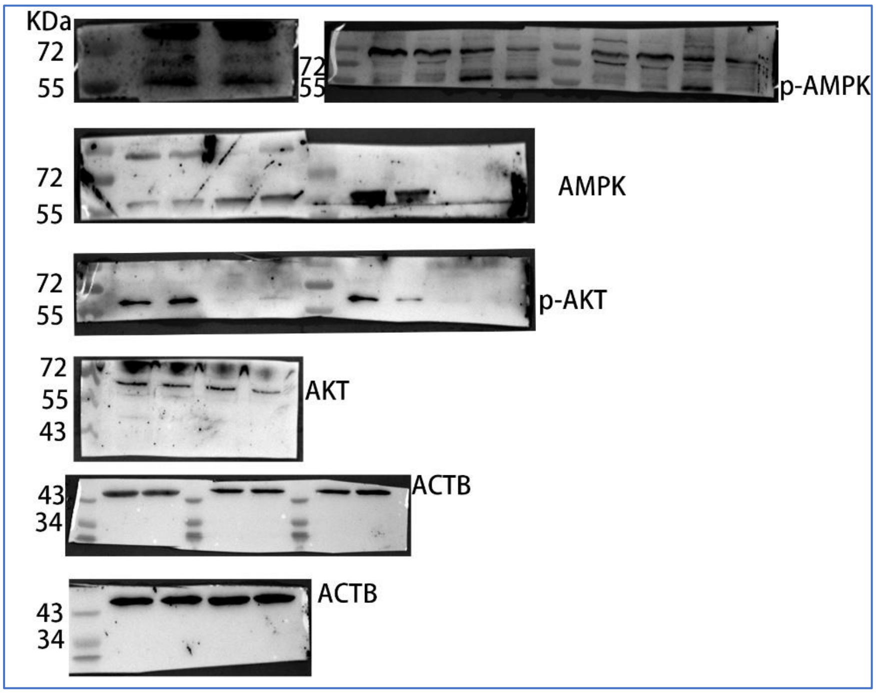

Supplementary Figure 22. Western blots of Figure 4c. The blot image is from seven blot membranes.

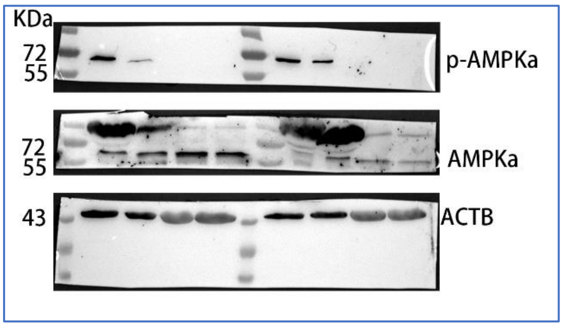

Supplementary Figure 23. Western blots of Figure 4f. The blot image is from three blot membranes.

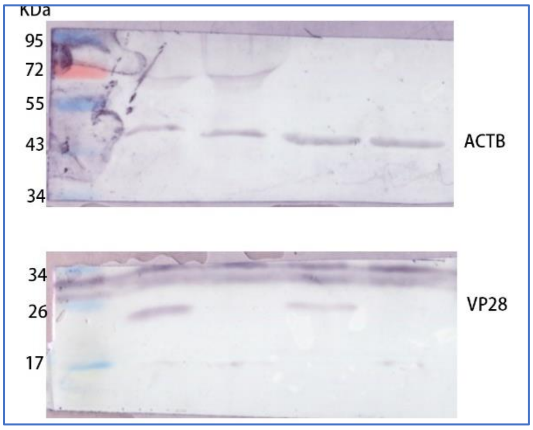

Supplementary Figure 24. Western blots of Figure 4h. The blot image is from two blot membranes.

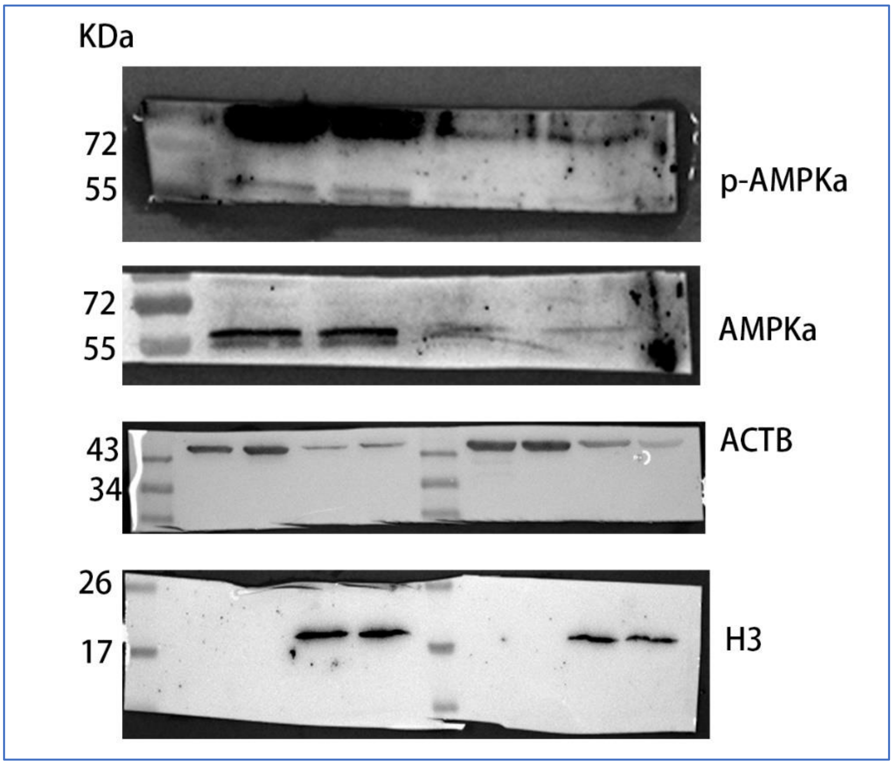

Supplementary Figure 25. Western blots of Figure5b. The blot image is from four blot membranes.

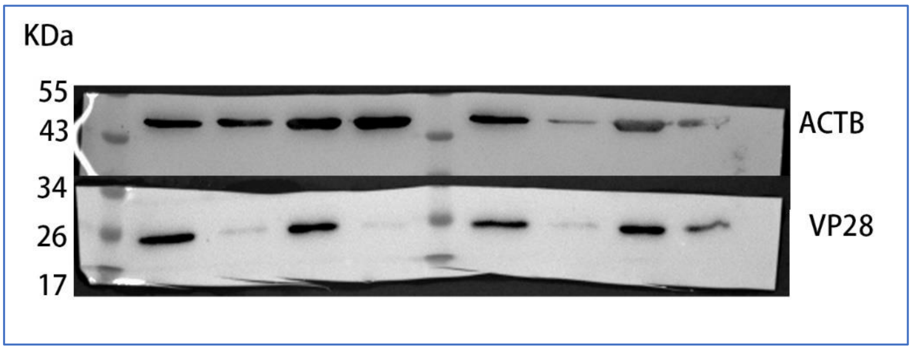

Supplementary Figure 26. Western blots of Figure 6c. The blot image is from the same blot membrane.

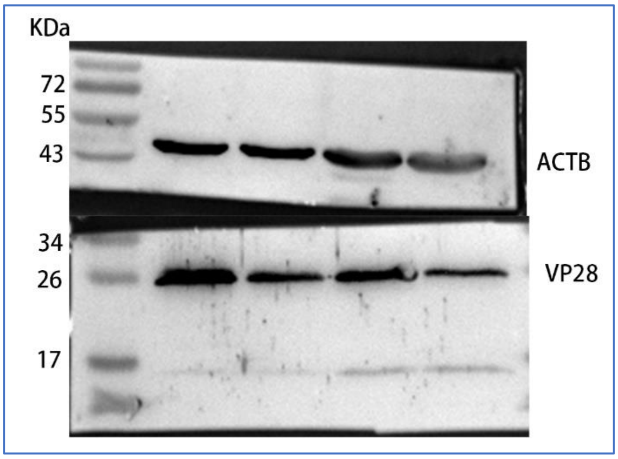

Supplementary Figure 27. Western blots of Figure 6h. The blot image is from the same blot membrane.

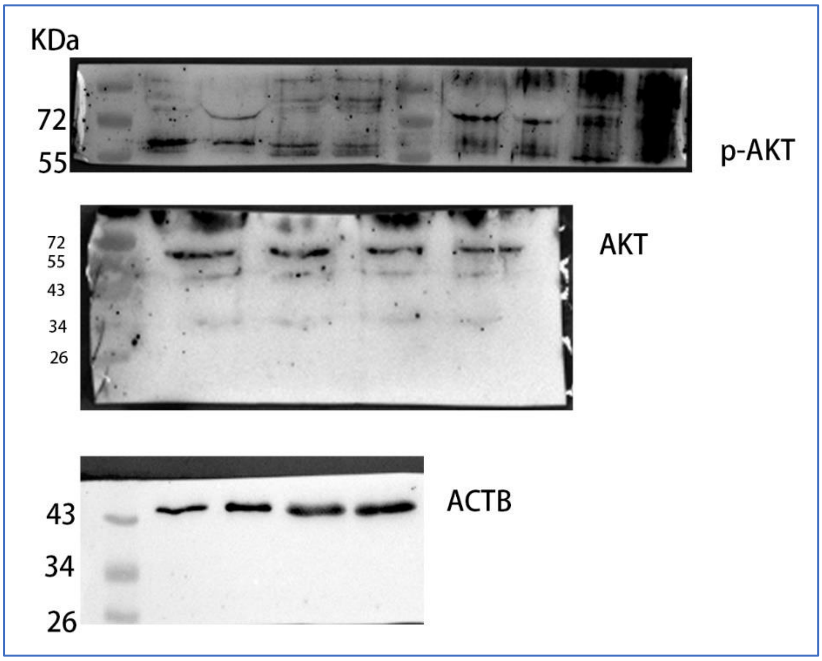

Supplementary Figure 28. Western blots of Figure 7a. The blot image is from three blot membranes.

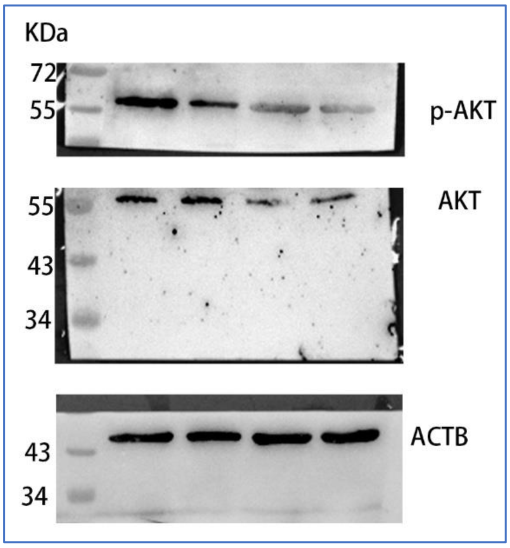

Supplementary Figure 29. Western blots of Figure 7b. The blot image is from three blot membranes.

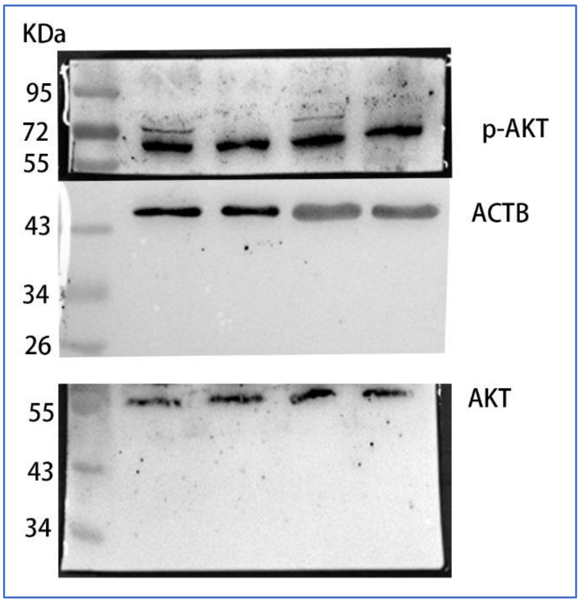

Supplementary Figure 30. Western blots of Figure 7c. The blot image is from three blot membranes.

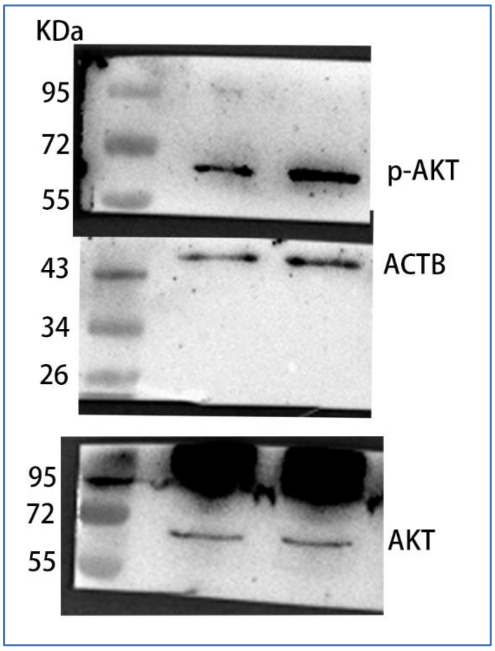

Supplementary Figure 31. Western blots of Figure 7d. The blot image is from three blot membranes.

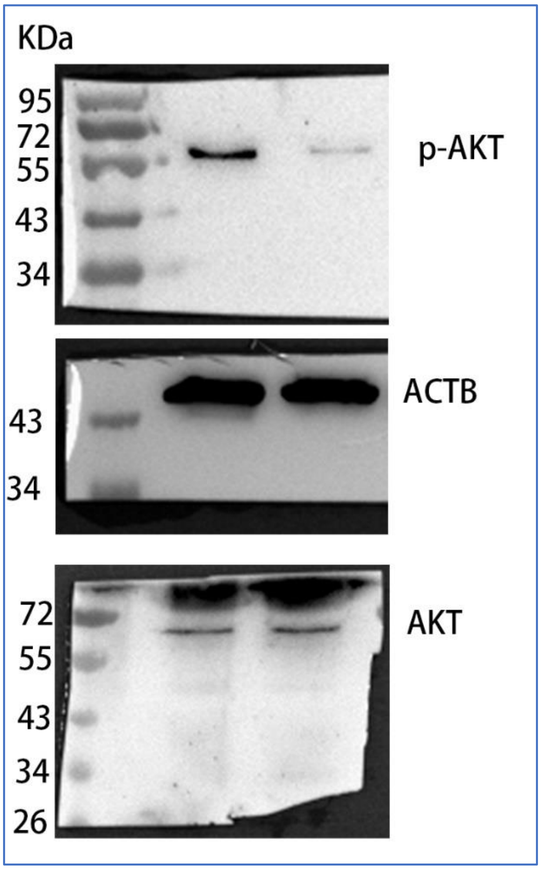

Supplementary Figure 32. Western blots of Figure 7e. The blot image is from three blot membranes.

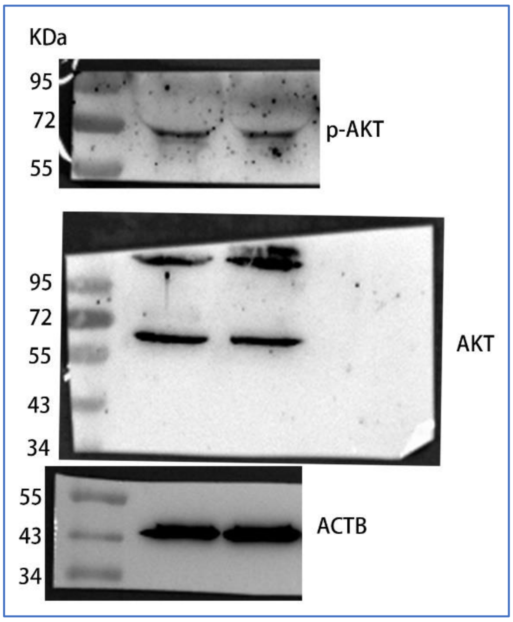

Supplementary Figure 33. Western blots of Figure 7f. The blot image is from three blot membranes.
