# Supplementary material for: WSSV exploits AMPK to activate mTORC2 signaling for proliferation by enhancing aerobic glycolysis
Source: Commun Biol. 2023 Apr 3;6:361. doi: 10.1038/s42003-023-04735-z (PMC10070494; doi:10.1038/s42003-023-04735-z)
Supplement: Supplementary file 2 — Description of Additional Supplementary Files [file 42003_2023_4735_MOESM2_ESM.pdf]

Description of Additional Supplementary Files

File name: Supplementary Data 1

Description: The numerical source data for graphs and charts in the paper.
