# Supplementary material for: WSSV exploits AMPK to activate mTORC2 signaling for proliferation by enhancing aerobic glycolysis
Source: Commun Biol. 2023 Apr 3;6:361. doi: 10.1038/s42003-023-04735-z (PMC10070494; doi:10.1038/s42003-023-04735-z)
Supplement: Supplementary file 4 — Reporting Summary [file 42003_2023_4735_MOESM4_ESM.pdf]

nature portfolio

Corresponding author(s): Jin-Xing Wang
Last updated by author(s): 2/27/2023

Reporting Summary

Nature Portfolio wishes to improve the reproducibility of the work that we publish. This form provides structure for consistency and transparency in reporting. For further information on Nature Portfolio policies, see our Editorial Policies and the Editorial Policy Checklist.

Statistics

For all statistical analyses, confirm that the following items are present in the figure legend, table legend, main text, or Methods section.

n/a	Confirmed	
☐	☒	The exact sample size (n) for each experimental group/condition, given as a discrete number and unit of measurement
☐	☒	A statement on whether measurements were taken from distinct samples or whether the same sample was measured repeatedly
☐	☒	The statistical test(s) used AND whether they are one- or two-sided<br>*Only common tests should be described solely by name; describe more complex techniques in the Methods section.*
☒	☐	A description of all covariates tested
☐	☒	A description of any assumptions or corrections, such as tests of normality and adjustment for multiple comparisons
☐	☒	A full description of the statistical parameters including central tendency (e.g. means) or other basic estimates (e.g. regression coefficient) AND variation (e.g. standard deviation) or associated estimates of uncertainty (e.g. confidence intervals)
☐	☒	For null hypothesis testing, the test statistic (e.g. F, t, r) with confidence intervals, effect sizes, degrees of freedom and P value noted<br>*Give P values as exact values whenever suitable.*
☒	☐	For Bayesian analysis, information on the choice of priors and Markov chain Monte Carlo settings
☒	☐	For hierarchical and complex designs, identification of the appropriate level for tests and full reporting of outcomes
☒	☐	Estimates of effect sizes (e.g. Cohen's d, Pearson's r), indicating how they were calculated

Our web collection on statistics for biologists contains articles on many of the points above.

Software and code

Policy information about availability of computer code

Data collection	Olympus BX51 fluorescence microscope (Shinjuku-ku, Tokyo) was used to acquire images of immunocytochemistry.<br>For collection of western blot data, HP scanner was used.<br>For collection of the agarose gel electrophoresis data, BIO-RAD Gel Doc Image system was used.<br>A microplate reader (TECAN M200 PRO, Tecan Group Ltd., Männedorf, Switzerland) was used calcium ions concentration, enzyme activity, and glycolysis metabolites.
Data analysis	MEGA 5.0 software was used for phylogenetic analysis.<br>MEGA 5.0, Genedoc and DNAMAN 6.0 software was used for the sequence alignment.<br>GraphPad Prism 8.0 software was used for data analysis and figure construction of the survival rate assay, the relative expression pattern.<br>Sequence similarity analysis was performed using BLAST.<br>The deduced amino acid sequences, molecular weight calculation, and isoelectric point analysis were carried out using ExPASy.<br>Protein domain prediction was performed using SMART.<br>Quantity One software was used for data analysis of the western blot figures.

For manuscripts utilizing custom algorithms or software that are central to the research but not yet described in published literature, software must be made available to editors and reviewers. We strongly encourage code deposition in a community repository (e.g. GitHub). See the Nature Portfolio guidelines for submitting code & software for further information.

Data

Policy information about availability of data

All manuscripts must include a data availability statement. This statement should provide the following information, where applicable:

- Accession codes, unique identifiers, or web links for publicly available datasets
- A description of any restrictions on data availability
- For clinical datasets or third party data, please ensure that the statement adheres to our policy

All data generated or analyzed in the study are included in this published article and its supplementary information files. The protein and cDNA sequences in the study were deposited in GenBank:The GenBank accession number of Ampka is OL364937, GenBank accession number of Ampkb is OL364938, GenBank accession number of Ampkr is XP042865543, GenBank accession number of Camkk is XP042859763, GenBank accession number of Hk is OL364939, GenBank accession number of Pfk is OL364940, GenBank accession number of Pk is OL364941, GenBank accession number of Glut1 is OL364942, GenBank accession number of Hif1a is XP042868035, GenBank accession number of Rictor is OK143319, GenBank accession number of Akt is KP419299, Dorsal is AME17867, and Relish is QPB70448. The plasmids used in this study are readily available upon request.

Human research participants

Policy information about studies involving human research participants and Sex and Gender in Research.

Reporting on sex and gender	no.
Population characteristics	no.
Recruitment	no.
Ethics oversight	no.

Note that full information on the approval of the study protocol must also be provided in the manuscript.

Field-specific reporting

Please select the one below that is the best fit for your research. If you are not sure, read the appropriate sections before making your selection.

☒ Life sciences ☐ Behavioural & social sciences ☐ Ecological, evolutionary & environmental sciences

For a reference copy of the document with all sections, see nature.com/documents/nr-reporting-summary-flat.pdf

Life sciences study design

All studies must disclose on these points even when the disclosure is negative.

Sample size	Three independent biological experiments ere performed in the study to ensure the findings are reproducible, and for statistical significance analysis.<br>For the survival rate analysis, seventy kuruma shrimp were separated into two groups: AICAR-injection and DMSO-injection (control). Each group contained 35 shrimp.<br>For the tissue sample collection, at least three shrimp were used in each group to erase the individual differences among shrimp.
Data exclusions	No data were excluded from the analyses
Replication	The measures were taken from three independent biological repeats to verify the reproducibility of the experimental findings. All the attempts at replication were successful.
Randomization	The experimental animals were selected randomly from each group.
Blinding	The investigators were blinded to group allocation during data collection for some experiments, such as gene sequencing and microscope data. For some experiments that the investigators are not blinded, such as expression pattern analysis, RNAi assays, survival rate analysis. This did not influence the outcomes of datum analysis because the experiments were performed in parallel and with statistical analysis.

Reporting for specific materials, systems and methods

We require information from authors about some types of materials, experimental systems and methods used in many studies. Here, indicate whether each material, system or method listed is relevant to your study. If you are not sure if a list item applies to your research, read the appropriate section before selecting a response.

Materials & experimental systems

n/a	Involved in the study
☐	☒ Antibodies
☒	☐ Eukaryotic cell lines
☒	☐ Palaeontology and archaeology
☐	☒ Animals and other organisms
☒	☐ Clinical data
☒	☐ Dual use research of concern

Methods

n/a	Involved in the study
☒	☐ ChIP-seq
☒	☐ Flow cytometry
☒	☐ MRI-based neuroimaging

Antibodies

Antibodies used	The following antibodies are prepared in our loboratory: Rabbit antiserum against Ampka；Rabbit antiserum against IMD; Rabbit antiserum against β-Actin;<br>The followings are commercial sntibodies:<br>anti-Histone-3 polyclonal antibodies (A2348, ABclonal, Wuhan, China); anti-phosphorylated (p)-AKT polyclonal antibodies (WLP001a, Wanleibio, Shenyang, China), AKT (WL0003b, Wanleibio, Shenyang, China), and anti-pAMPKα (YP0575, ImmunoWay, Plano, TX, USA), the secondary antibody, goat anti-rabbit antibody conjugated with alkaline phosphatase (ZB2308 ZSGB-Bio, Beijing, China)
Validation	The following antibodies are prepared in our loboratory: Rabbit antiserum against Ampka；Rabbit antiserum against IMD; Rabbit antiserum against β-Actin;<br>The followings are commercial sntibodies:<br>anti-Histone-3 polyclonal antibodies (A2348, ABclonal, Wuhan, China); anti-phosphorylated (p)-AKT polyclonal antibodies (WLP001a, Wanleibio, Shenyang, China), and anti-pAMPKα (YP0575, ImmunoWay, Plano, TX, USA), the secondary antibody, goat anti-rabbit antibody conjugated with alkaline phosphatase (ZB2308 ZSGB-Bio, Beijing, China)

Animals and other research organisms

Policy information about studies involving animals; ARRIVE guidelines recommended for reporting animal research, and Sex and Gender in Research

Laboratory animals	Healthy kuruma shrump, M .japonicus (8-12 g each) were obtained from a shrimp farm in Qingdao, Shandong Province, China. Shrimp were cultured in a recirculating aquaculture system filled with natural seawater at a salinity of about 2.2% (w/v) at 25 degree centigrade. Shrimp were fed with a commercial diet daily, and the experimental animals were selected from each group.
Wild animals	The study does not involve wild animals.
Reporting on sex	The sex of the prawns was not distinguished in the study.
Field-collected samples	The study does not involve animals captured from the field.
Ethics oversight	The antibody preparation using rabbits was conducted under the protocols approved by the ethics committee in School of Life Sciences, Shandong University.

Note that full information on the approval of the study protocol must also be provided in the manuscript.
